# An assistive robot that enables people with amyotrophia to perform sequences of everyday activities

**DOI:** 10.1038/s41598-025-89405-2

**Published:** 2025-03-11

**Authors:** Annette Hagengruber, Gabriel Quere, Maged Iskandar, Samuel Bustamante, Jianxiang Feng, Daniel Leidner, Alin Albu-Schäffer, Freek Stulp, Jörn Vogel

**Affiliations:** Institute of Robotics and Mechatronics, German Aerospace Center (DLR), Oberpfaffenhofen, Wessling, 82234 Germany

**Keywords:** Assistive robot, Manipulation aid, Re-enabling, Intuitive task execution, sEMG-based interface, Shared control, Whole body control, Activities of daily living, Engineering, Health care

## Abstract

Mobile manipulation aids aim at enabling people with motor impairments to physically interact with their environment. To facilitate the operation of such systems, a variety of components, such as suitable user interfaces and intuitive control of the system, play a crucial role. In this article, we validate our highly integrated assistive robot EDAN, operated by an interface based on bioelectrical signals, combined with shared control and a whole-body coordination of the entire system, through a case study involving people with motor impairments to accomplish real-world activities. Three individuals with amyotrophia were able to perform a range of everyday tasks, including pouring a drink, opening and driving through a door, and opening a drawer. Rather than considering these tasks in isolation, our study focuses on the continuous execution of long sequences of realistic everyday tasks.

## Introduction

Stroke, spinal cord injury, and neuromuscular diseases often result in permanent motor impairment and subsequent disability. In 2013, approximately 5.4 million people in the United States were affected by paralysis, of which the majority (72.1$\%$) was younger than 65 years^1^. Performing Activities of Daily Living (ADL) can become challenging – even impossible – with motor impairment, and may lead to a dependence on caregivers for everyday life. Assistive Technologies (ATs) enable those affected to perform such tasks independently. For instance, for people with paralysis, a power wheelchair with a robotic manipulator provides mobility and the ability to physically interact with the environment. Power wheelchairs are widespread and can be steered easily by a 2-degrees of freedom (DoF) joystick. In comparison, a robotic manipulator has many DoFs – six for the end effector (EE) pose plus at least one for the tool. Controlling all available DoFs of such ATs with a lower-dimensional interface is challenging and often achieved through ‘mode switching’, in which the user selects different subsets of DoFs to be directly controlled.

**Fig. 1:**
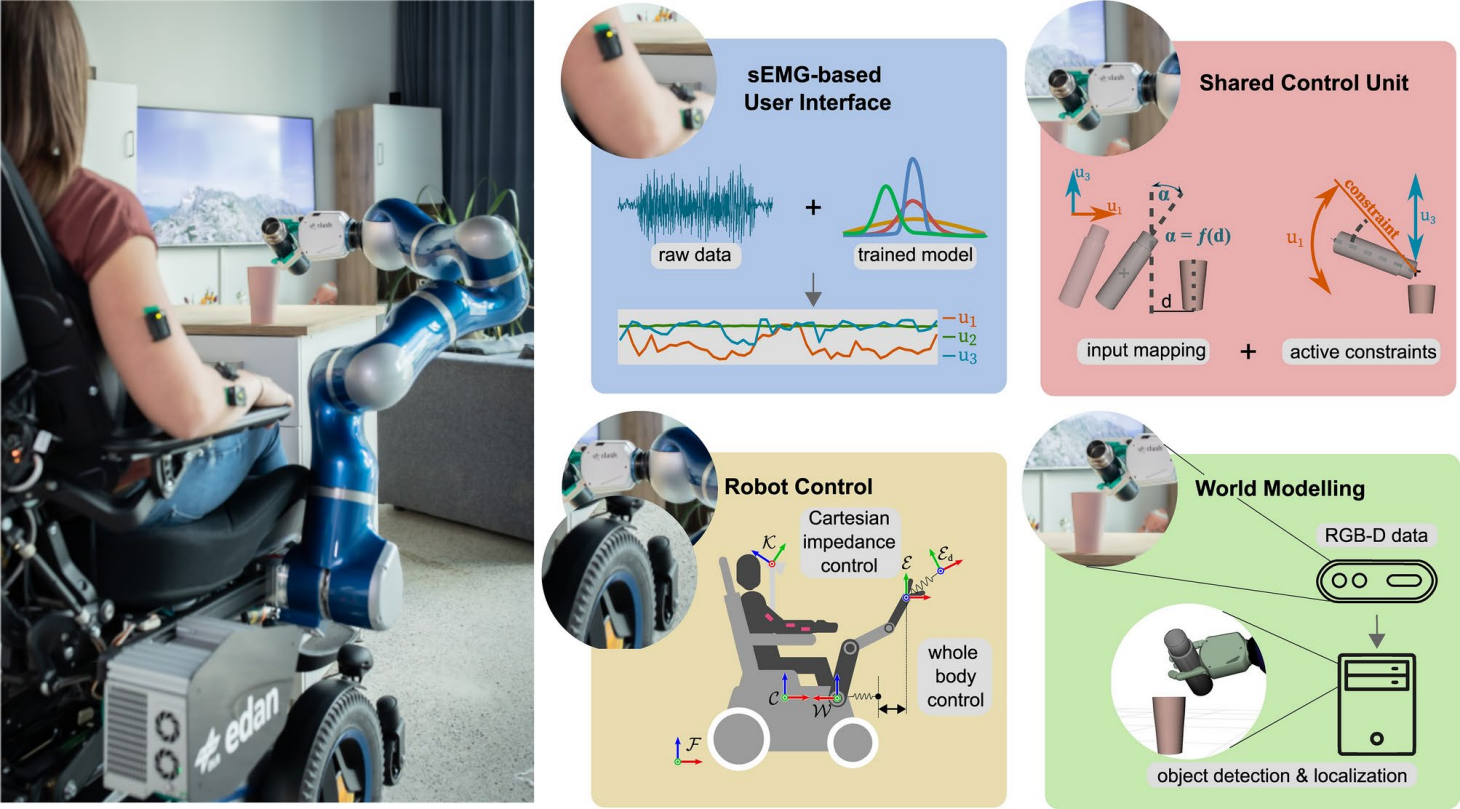
An overview of the EDAN system, illustrating the main building blocks needed to provide people with severe motor impairment with intuitive control in everyday tasks. This includes *(in blue)* the interface based on surface electromyography (sEMG), which enables people with severe amyotrophia to perform 3D robot control, *(in red)* a shared control scheme based on Shared Control Templates which supports the user during complex tasks, *(in green)* the world modeling which provides information about the surrounding objects, and *(in yellow)* the robot control unit for safe and coordinated motion of the arm and the platform.

People in need of such an assistive device often do not have the ability to operate a mechanical 2-DoF joystick. Apart from commercially available alternatives, such as chin-joysticks or sip-and-puff switches, different solutions for 3D robot control are being investigated^2^, including Brain-Computer Interfaces (BCI), where control commands are extracted from brain signals. Electroencephalography (EEG) is the most common approach in non-invasive BCIs^3,4^. However, controlling systems with many DoFs via EEG poses challenges such as long training time, user fatigue, and low signal-to-noise-ratio^5^. Invasive BCIs offer advantages over non-invasive ones, such as providing higher signal bandwidth and higher spatial resolution^6^. The first use of invasive BCIs for continuous 3D robot control in paralyzed individuals was presented by Brown University and DLR in Hochberg et al.^7^, followed by Collinger et al., who achieved 7-DoF control^8^. Recent findings suggest that incorporating tactile feedback can enhance BCI control^9^.

Other bioelectrical signals, such as muscular activity, can also be used as control interfaces for ATs. Surface Electromyography (sEMG) – the measurement of muscular activity – is used in a wide range of applications including myoelectrical prosthesis^10^, rehabilitation^11^, computer gaming^12^, and teleoperation for space applications^13^. sEMG has also been used to create control interfaces for robotic manipulators for non-disabled users^14–16^. Seeing the potential of this technology for people with motor impairments, we developed an sEMG-based interface that extracts 3D continuous control commands from residual muscular activity using Gaussian process regression^17^. With this interface, we showed that two individuals, who are no longer able to move their limbs due to severe spinal muscular atrophy (SMA), were able to control a stationary robotic arm^18^.

Independent of the input device, the coordination of many DoFs – as in a mobile assistive robot composed of wheelchair, arm, and gripper – is challenging for humans. An integrated assistance during task execution would help to overcome this challenge. Research has shown that people with disability do not necessarily prefer a high level of robot autonomy^19^, but rather want to be empowered by being in control of the system’s actions^20^. This can be achieved with support via shared control approaches. Shared control can assist the user in controlling the robot with different modalities, e. g. at the force level^21^, with policy blending^22^, user input modulation^23^, or task space restrictions^24,25^. Modeling of the user’s intent has also been investigated: Naughton et al. propose structured action predictions for dynamic environments, learned from demonstrations^26^. User intent is also used to adjust the level of command blending^22,27,28^. Gopinath et al. influence the control mode selection to maximally disambiguate user intent^29^, while others apply intent inference to restrict the task space^30–32^.

Our approach, called ‘Shared Control Templates’ (SCTs)^33^, simplifies robot control by mapping user commands to task-relevant motions and by enforcing geometric task space constraints. Thus the system supports the user during complex robot motions, e.g. motions with combined translations and rotations, whilst ensuring that the user stays in command at all times. Switching between different control schemes becomes also obsolete with this method. On top of the SCTs, we have integrated whole-body control (WBC)^34,35^ into the low-level control system, which automatically couples the DoFs of the wheelchair and arm based on the specifics of a task. Using this approach, ADLs with extended ranges of motion, such as opening drawers or doors, can be performed fluently, without switching between the wheelchair and robotic arm ^33,36^.

To make mobile ATs, as shown in Fig. 1, more efficient, versatile, and empowering, the aforementioned technologies necessitate integration into a holistic system capable of operating in a self-sufficient way. Furthermore, evaluation with people from the target group is essential to prove that new developments bring assistance to those who need it. Table 1 gives an overview of research studies on assistive robotic manipulators that include evaluations with users from the target group. Intuitive interfaces or shared-control approaches have been investigated in multiple studies, e.g. during feeding^20,37,38^, or other ADLs^39^. Diverse input devices such as BCIs^40^ or remote teleoperation devices^41–43^ were also investigated. However, existing work typically investigates specific features or tasks in isolation. Entire systems and sequences of tasks, as they appear in everyday life, such as pouring and drinking in succession, are rarely considered.

We developed EDAN the 'EMG-controlled Daily AssistaNt’^44^, a unified mobile manipulation aid designed to address all aforementioned challenges in one integrated system, achieving robust task execution with intuitive control. The primary contribution of this article is the validation of our system through a case study involving individuals from the target group. Specifically, we demonstrate the feasibility and effectiveness of our system by showing that people with severe amyotrophy can use the sEMG-based interface with SCT support to perform realistic sequences of everyday tasks. This case study presents three users successfully completing tasks such as pouring, drinking, and opening drawers using our assistive robot EDAN. Despite limited experience with the system, participants controlled the entire system by themselves during task execution.

**Table 1:** Overview of selected research on assistive robotic manipulation with users with impairments since 2011.

	Hochberg et al.^7^	Collinger et al.^8^	Flesher et al.^9^	Kim et al.^19^	Bhattacharjee et al.^20^	Muelling et al.^40^	Losey et al.^23^	Ciocarlie et al.^41^	Javaremi et al.^39^	Park et al.^37^	Ding et al.^45^	Morbidi et al.^46^	Erdogan et al.^47^	this work
Users with impairment	$\bullet$	$\bullet$	$\bullet$	$\bullet$	$\bullet$	$\bullet$	$\bullet$	$\bullet$	$\bullet$	$\bullet$	$\bullet$	$\bullet$	$\bullet$	$\bullet$
Mobile assistive robot					$\bullet$			$\bullet$		$\bullet$		$\bullet$	$\bullet$	$\bullet$
Continuous interface	$\bullet$	$\bullet$	$\bullet$	$\bullet$		$\bullet$	$\bullet$		$\bullet$		$\bullet$	$\bullet$	$\bullet$	$\bullet$
Assisted control scheme				$\bullet$	$\bullet$	$\bullet$	$\bullet$	$\bullet$		$\bullet$	$\bullet$	$\bullet$	$\bullet$	$\bullet$
Direct control scheme	$\bullet$	$\bullet$	$\bullet$	$\bullet$		$\bullet$		$\bullet$	$\bullet$		$\bullet$	$\bullet$	$\bullet$	$\bullet$
Both schemes combined								$\bullet$			$\bullet$		$\bullet$	$\bullet$
Whole-body control														$\bullet$
Pick/place	$\bullet$	$\bullet$	$\bullet$	$\bullet$		$\bullet$	$\bullet$	$\bullet$	$\bullet$		$\bullet$			$\bullet$
Utilizing objects			$\bullet$		$\bullet$	$\bullet$	$\bullet$	$\bullet$	$\bullet$	$\bullet$	$\bullet$			$\bullet$
Drinking	$\bullet$										$\bullet$			$\bullet$
Feeding		$\bullet$			$\bullet$		$\bullet$		$\bullet$	$\bullet$				
Articulated objects								$\bullet$	$\bullet$					$\bullet$
User mobility												$\bullet$	$\bullet$	$\bullet$

The first block shows the technological features enabling the research, the second block classifies the tasks that have been performed in the user studies. Note that in this overview the work of Morbidi et al. and Erdogan et al.^46,47^ are two representatives of research on assistive wheelchairs without manipulation capabilities.

## Results

### Experimental conditions

As shown in Fig. 1, EDAN integrates high- and low-level control modules as well as a suitable user interface based on bioelectrical signals. The system is commanded with continuous velocity signals and a binary trigger signal. A mechanical head switch is used to cycle through the controllable devices: *robotic arm*, *wheelchair*, *tablet* and *nothing*. On the tablet, a Graphical User Interface (GUI) provides information about the state of the system, the control schemes, the output of the command interface, and the tasks which can be supported by the system. This GUI also enables the user to switch between different control schemes, such as direct control and shared control by activating the sEMG-based trigger.

In total, three participants – whom we refer to as P-A, P-B and P-C – participated in five to six sessions each, with the aim to perform a long sequence of everyday activities in the last session. To familiarize participants with the system, the complexity of the performed tasks was increased along sessions. The first session consisted of wheelchair adjustments and electrodes placement. From the second session onward, participants actively controlled the system. This resulted in four or five experimental sessions for each participant to actively use our system with a experiment time of approximately 1.5 hours per session. Because no qualified caregiver was available to transfer P-B to the wheelchair, P-B performed the fourth session lying next to EDAN. For the same reason, session six could not be realized for P-B, which is why P-B was not able to perform the full sequence of tasks in one go. P-A and P-B conducted the experiments in their apartments using unmodified objects of daily living, while P-C conducted experiments in a kitchen environment set up in our laboratory. For details see Supplementary Materials (SM) 1.2.

P-A and P-B used the sEMG-based interface with 3D continuous commands plus the trigger being decoded from residual muscular activity. P-C is capable of using a 2-DoF joystick and controlled the system with a hybrid interface, where sEMG is used in addition to the joystick for the third dimension (vertical movements) and a trigger signal.

### EDAN enabled participants to perform activities of daily living

In their last session, P-A and P-C could complete a sequence of tasks, namely fetching a mug from a drawer, pouring water from a bottle into the mug, and drinking from it. To successfully execute the sequence of tasks, the full functionality of the EDAN system had to be used by the participants: the sEMG-based interface, the GUI, and the control schemes of direct control (DC), shared control (SC), and shared control with whole-body control (SC-WBC), as well as driving the wheelchair in direct control (W-DC). Table 2 shows the whole sequence P-A and P-C performed with EDAN, including times needed for the sub-tasks. It is important to note that participants were asked to perform the tasks in succession without instructions to be particularly fast in task completion.

**Table 2:** Tasks performed by P-A and P-C in one sequence.

	Sub-task	Control scheme	P-A	P-C
1	Open a drawer	SC-WBC	23 s	35 s
2	Relocate wheelchair	W-DC	20 s	21 s
3	Pick a mug from the drawer	DC	50 s	88 s
4	Relocate wheelchair	W-DC	0 s	10 s
5	Close the drawer	DC	25 s	23 s
6	Relocate wheelchair	W-DC	11 s	0 s
7	Place the mug on top of the cabinet	DC	75 s	30 s
8	Pick a bottle	SC	37 s	26 s
9	Pour liquid into the mug	SC	48 s	26 s
10	Place the bottle	SC	9 s	8 s
11	Pick the mug	SC	35 s	9 s
12	Drink from the mug	SC	44 s	6 s
13	Place the mug back on the table	SC	12 s	34 s
	Time for transitions	–	170 s	140 s
		Total	9:20 min	7:48 min

In total P-A needed 9:20 min and P-C 7:48 min to perform the given sequence. For each subtask the control scheme and duration are given. For SC-tasks the time illustrates the duration when the control scheme was activated. This was triggered by the task inference module, which activated the task with highest probability, including the distance between EE and the object. For DC-tasks a visual estimation on the beginning and the end of each subtask was made. W-DC indicates direct control of the wheelchair and could be used freely by the participants. Thus, 0 s indicates that the subject did not move the wheelchair at this time. ‘time for transitions‘ is the accumulated time of tablet use or if no task was active.

**Fig. 2:**
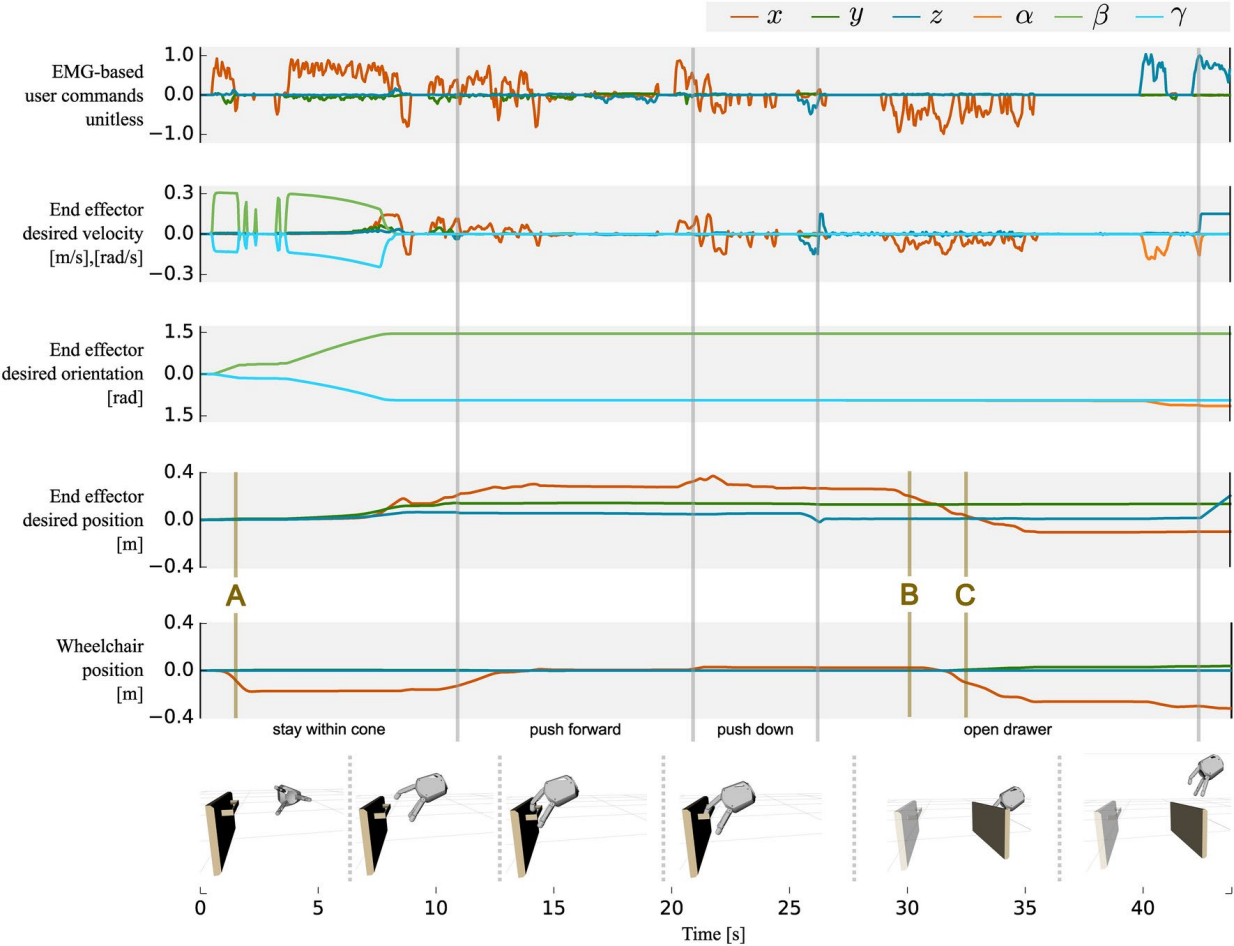
Modulation of the sEMG-input to EE and wheelchair motions during the opening of a drawer performed by P-A. The task consists of five SCT states, including ‘stay within cone’, ‘push forward’, ‘push down’, and ‘open drawer’. The first row shows the user commands **u** decoded from measured muscular activity. The second row shows the desired velocity commands resulting from the commands **u** combined with the input mapping and active constraints from the SCT. The orientation and position of the EE are shown in the third and fourth row, respectively. The fifth row shows the commanded wheelchair trajectory. Letters **A-C** illustrate the use of whole-body control. (**A**) While the constraints of the first state leads to an EE rotation, the workspace limits are reached and the wheelchair moves back to increase the workspace to perform the task. (**B**) As long as the workspace is sufficient while pulling the drawer, only the EE moves backwards. (**C**) As soon as the workspace limits of the EE are reached, the wheelchair moves back automatically.

**Fig. 3:**
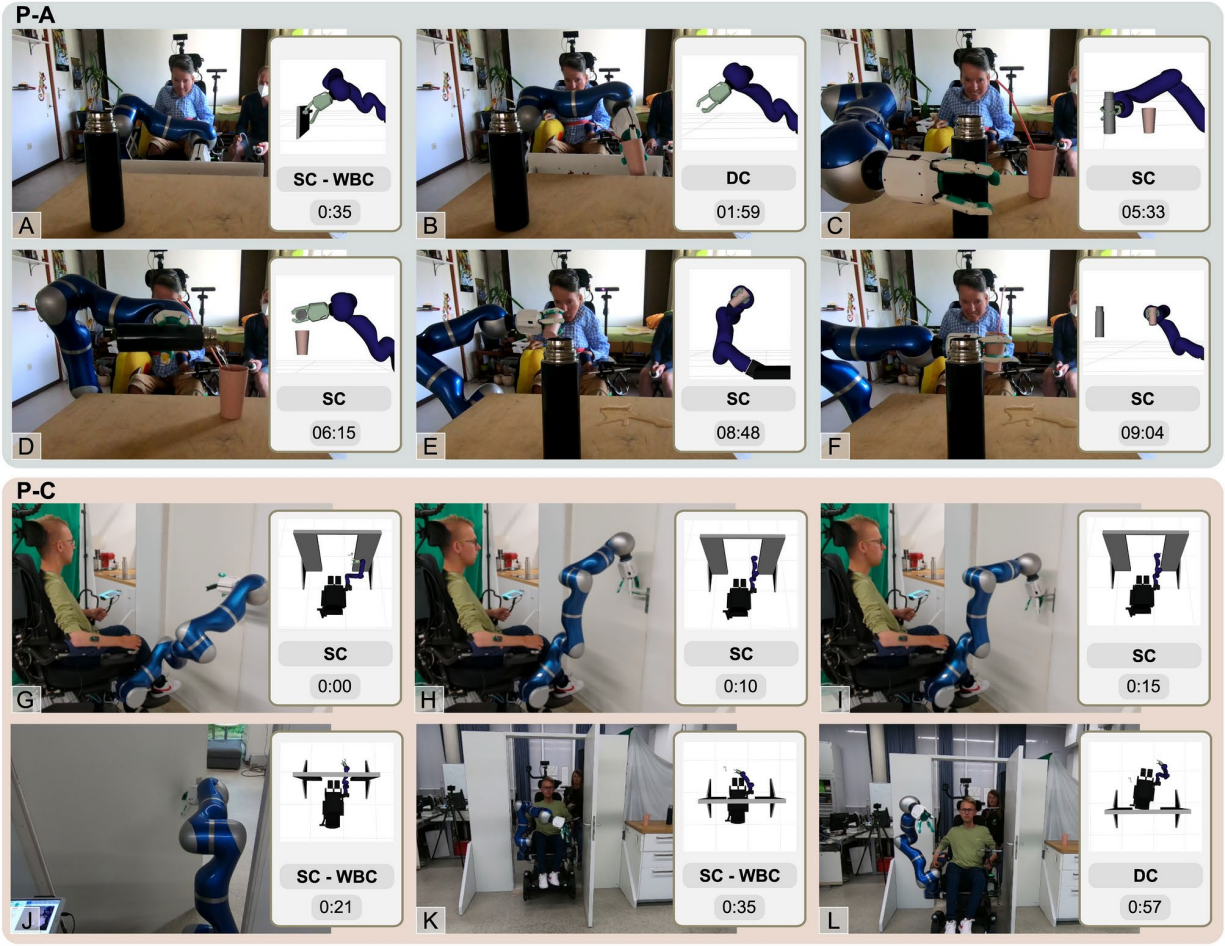
Photo series of the execution of different ADLs, using the EDAN system. The upper part shows P-A performing the sequence of tasks. For each sub-figure the robot world model, the control scheme, and the time stamps are visualized. (**A**) Open the drawer using SC-WBC. (**B**) Pick a mug from the drawer using DC. (**C**) Pick a bottle with SC. (**D**) Pour into the mug with SC. (**E**) Drink from the mug using SC. (**F**) Release the mug, again with SC. The lower part shows P-C using the hybrid interface to open a door and drive through. The robot world model, the control scheme and the time stamps are visualized here as well. (**G**) Start of the SC-task, with the robotic arm next to the handle. (**H**) The user is guided by the SCT to place the robotic EE above the door handle. (**I**) Pressing the door handle. (**J**) Open the door along a circular motion, while the wheelchair is following through the door with WBC. (**K**) Release the door handle. (**L**) Drive the remaining path through the door, using DC.

P-A and P-B performed in their fourth session the SCT-based subset of tasks 8-13 in 4:15 min and 3:39 min, respectively. In her sixth session, P-A performed the entire sequence using EDAN in 9:20 min, as illustrated in Fig. 3 and in Video 2 in the SM. P-C performed the whole sequence controlling EDAN with the hybrid sEMG-based interface in 7:48 min during his fifth session, shown in Video 1 in the SM. In sub-task 9 ‘pour liquid into the mug’, P-A used the freedom given by the SCTs and explored the direction of pouring along an arc around the mug (only the DoFs that are relevant for task success i.e. height and orientation are constrained), resulting in 48 s for this sub-task. The drinking task (12) was two-fold: first, bringing and tilting the mug close to the user’s head, supported by shared control, while the straw used for drinking had to be placed by the participants by controlling the 2 DoF of the **y**-**z**-plane. Due to the lack of head motion capabilities, P-A took 7 times longer than P-C, as the straw had to be placed exactly in front of her mouth.

Figure 2 illustrates the tasks of opening a drawer, which depicts the benefit of shared control approaches when controlling more DoFs than the interface can address. To position the robotic hand in front of the drawer handle, careful coordination between translational and rotational movements is necessary. The phase ‘stay within cone’ shows that the participant provided input only in **+x** direction (towards the drawer), while the EE aligned itself in position and orientation w. r. t. the handle.

Furthermore, the opening of the drawer required a large linear motion of the EE, despite a very limited manipulator workspace due to safety constraints (the manipulator must never enter the space occupied by the user’s legs). This workspace limitation was addressed with WBC. At task start, the wheelchair initially moved backwards, as shown in Fig. 2 annotated with **A**. Parameterized from the SCT, WBC automatically determined that a wheelchair motion was necessary given the insufficient workspace to achieve the desired EE motion, and computed the required coupling between the wheelchair and manipulator DoFs. During the phase ‘open drawer’ the user gives commands in **-x** direction to pull open the drawer. As long as the workspace is sufficient to move the EE back, only the manipulator is moving (marked with **B**). As soon as the workspace limits are reached, the wheelchair moves backwards as well to continue performing the task. This automatic coordination can be observed in Fig. 2 at position **C**. Switching between wheelchair and manipulator control was thus not necessary for this task. Furthermore, the user was still in control during SCT-tasks, e. g. deciding how far should the drawer be opened.

### EDAN enables mobility in everyday life

Besides this sequence of everyday activities, P-C used EDAN to perform additional tasks, namely opening and driving through a door and opening a fridge. Both tasks have been executed during the fifth experimental session of P-C and can be found in Video 3 and 4 in the SM. The lower part of Fig. 3 shows P-C while opening and driving through the door within 57 sec. Supported by SCTs, P-C was able to open the door by giving commands roughly towards the door handle, without the need for mode switches or precise commands (Fig. 3 G–I). To keep the robotic arm within its reachable space, WBC automatically moved the wheelchair forward on a straight line through the door (Fig. 3 J). Within the task, wheelchair rotations were inhibited to safely travel through the door. Although this task is highly constrained, the participant was free to proceed with the task at their own pace or even move backwards within the task at any time. Once the door was fully opened, the participant exited the task by letting go of the door handle through moving the EE upwards. Passing through the door is then achieved using direct wheelchair control (Fig. 3 K–L).

## Discussion

In this article, we present a case study in which three participants with amyotrophia independently operate the robotic assistant EDAN, enabling them to complete sequences of tasks despite limited familiarization time. EDAN provides high usability facilitated through different control schemes such as direct control and shared control, and an sEMG-based interface for 3D robot control. Participants engaged in a realistic scenario of preparing and consuming a drink, involving multiple objects and different control schemes in one long sequence. Existing work often concentrates on a few tasks, evaluating specific features in isolation to quantify their performance (cf. Table 1). To advance ATs for everyday use, it is crucial to validate them with the actual target group, in realistic (home) environments, and on long sequences of relevant tasks representative of daily life (not only on isolated and artificial benchmark tests). Wheelchair-based assistive robots controlled by users with severe motor impairments have not, to our knowledge, achieved this level of performance on such complex and consecutive tasks.

Potentially, any interface providing three continuous DoFs and a binary trigger signal can be used to control EDAN. We showed that our sEMG-based interface enabled people with severe amyotrophia to perform ADLs by controlling all functions of the EDAN system, such as moving the robot, the wheelchair and operating the tablet GUI. P-A and P-B are suffering from an advanced SMA, leaving them without notable functional movements in the limbs. By encoding a 3D control signal and a trigger from residual muscular activity they were able to handle the system and to perform all given tasks. P-C commanded 1-DoF and the trigger via muscle signals, successfully augmenting a 2-DoF joystick. Although the times shown in Table 2 do not result from timed trials, it is notable that the handling of the robot by P-A and P-C is at a comparable level. This reveals that our interface based on muscular activity can be used in different settings and gives people with varying degrees of muscle atrophy the opportunity to realize continuous 3D robot control.

Nevertheless, different aspects have to be addressed before using our sEMG-based interface in everyday life. Arm position, electrode positioning, muscle fatigue, and involuntary muscle contractions can alter sEMG-signals and negatively influence the interface performance, especially in long term use. Online adaptation of the decoder^48^ or techniques such as transfer-learning^49–51^ could be used to stabilize interface performance and avoid daily decoder training.

Shared control was instrumental to successfully perform complex ADLs, such as pouring from a bottle, even with limited familiarization time with the system (four or five robot sessions with 1.5h of gross experimentation time). Participants could focus on handling the principal motions for the tasks and let the assistance take care of coordinated motions, resulting in simultaneous control of all relevant DoFs. Input mapping and active constraints made mode switching unnecessary and allowed for efficient and safe task execution. Completion time was not focused on in this study as it is known that time efficiency plays a subordinate role for people using robotic manipulation aids^52^. Hence, participants could explore their agency over the robot’s motions, e.g. P-A explored the pouring direction, resulting in a longer completion time for this sub-task.

Additionally, challenging ADLs like opening a door, which requires continuous contact with the handle and coordinated arm and wheelchair motion, could be performed by a participant in a short time. This was achieved through shared control with whole body control, enabling an effective and intuitive operation of the 17-DoF robotic system (wheelchair: 2, manipulator: 8, hand: 7). During SC-WBC the wheelchair and manipulator are considered as a single kinematic chain, removing the need for device switching within a task. This could also be observed in the sub-task ’opening a drawer’ of P-A where the wheelchair was moved twice automatically during the tasks. For comparison, the wheelchair was moved also twice by P-A during the DC tasks 1-7 of Table 2 at the beginning of the sequence, each time requiring switching the device to be controlled.

Being time-independent and object centric, one drawback of the current implementation of SCTs is the lack of consideration for kinematic limits and environmental constraints, as the assistance only depends on the current EE pose w. r. t. a known target object. To address this limitation, a novel task representation with task feasibility checks is being developed^53^, ensuring collision-free execution of tasks in cluttered environments. Another aspect is an accurate pose estimation of the tasks objects, as even small deviations can lead to poor execution of the tasks, such as spilling water, which has occurred in P-A’s experiments. An object tracking approach^54^ could be used to track dynamic or occluded objects, such as the grasped object, leading to higher precision in multi-object tasks, like during pouring. Furthermore, an correction mode should be implemented, allowing users to correct the assistance in specific phases of the task if needed. Finally, to scale to new objects and situations, SCTs should not be hand-coded, but rather partially learned from demonstrations, e. g. provided by remote teleoperation, kinesthetic teaching or direct control by the user, as investigated in^55,56^.

## Materials and methods

### Participants

In total three participants took part in this pilot study. P-A and P-B are two 52-year old women suffering from spinal muscular atrophy type II. Due to a strong progression of muscular atrophy, voluntary limb movements are hardly possible and both are dependent on 24-hour care. Nevertheless, both can evoke voluntary muscle activation at different locations along their arms. Despite the lack of function, these muscle contractions are still measurable with surface EMG. P-A and P-B used the full sEMG-based interface to conduct the experiment. An initial contact with the sEMG-based interface was made in a study in 2013^57^. Subsequent studies in 2017^18^ utilized the Action Research Arm Test (ARAT) with a direct control scheme to operate a robotic manipulator, followed by similar experiments conducted in 2019. Participant P-A and P-B collectively accumulated less than 10 hours of experiment time with the interface, spread across multiple years prior to this study. Additionally, the participants were not familiar with shared control implementation nor the entire system presented in this manuscript.

The evaluation using the hybrid interface was conducted by one male participant (P-C, age 24), suffering from Dystrophy Becker-Kiener (Type 43). His residual muscular activity only allows for limited upper limb movement, while tasks involving weight or requiring outstretched arms, such as drinking from a bottle or opening a door, can not be performed. In daily life, P-C uses a commercial 2-DoF joystick to move his wheelchair. He had no prior experience with the sEMG-based interface nor the EDAN system.

All participants gave written consent to the procedure, which was explained to them orally and in a written form. The study was conducted according to the guidelines of the Declaration of Helsinki, and approved by the Ethics Committee of the Technical University of Munich, School of Medicine (approval number: 6/14S). Furthermore, all participants gave their written consent to the publication of identifying information, such as images and videos, for scientific publications.

### Experimental procedure

The whole study consisted of six sessions for P-A and five sessions for P-B and P-C. Each session took approximately 3 hours in total, to not overload the participants cognitively and physically. During the first session, no experiments took place. This session was used to determine individual placement of sEMG sensors and to adapt the wheelchair to the individual needs of the participants. Furthermore, virtual workspace limitations for the robotic arm were specifically adjusted in accordance with the users position to prevent any contact between user and manipulator. All other sessions included the experiments and a preparation phase beforehand.

The preparation phase included several steps: sensor attachment, training the Gaussian process for the sEMG-based interface, and transfer to the wheelchair if applicable. For P-A and P-B, the attachment of the sEMG-sensors and training of the sEMG-decoder were done in bed instead of in the wheelchair, to spare limited sitting time for the actual tasks execution. We did not observe any disturbance on the interface performance resulting from the transfer from the bed to the wheelchair. When operating EDAN from outside the wheelchair, the robot was placed close to the participants bed, such that it was still possible for them to overview the scene from a lying position next to the system. The tablet, which provided the GUI, was mounted in their field of view, but not obstructing the workspace of the manipulator. While P-A and P-B conducted the experiments in their home environment, P-C conducted the experiments in a kitchen-like environment in our lab.

In total the experimental sessions took place as follows:

- P-A participated in five experimental sessions, sitting in EDAN in three of them.
- P-B participated in four experimental sessions, sitting in EDAN once.
- P-C participated in four experimental sessions, sitting in EDAN in all of them. 

See SM 1.2 for the detailed schedule of the sessions.

The gross time for the experiment was approximately 1.5 h, including explanations of the tasks, robot control to perform those tasks, and individual breaks that the participants needed. At the beginning of the experiments, the participants familiarized themselves with the interface and the system by performing pick-and-place tasks using direct and shared control. Individual tasks were explained and practiced in collaboration with the experimenter to explain the workings of the assistance. Each task was executed a maximum of three times before being performed in a sequence. Participants were further instructed to perform the given tasks in succession but not to execute them as quickly as possible. During evaluations, no verbal instructions were given. Participants were given verbal feedback only upon request, to confirm the task sequence order if needed.

### The EDAN system

The robotic system EDAN^44^ is composed of a commercially available wheelchair equipped with an 8-axis version of the DLR Light Weight Robot III. The additional revolute joint at the base of the robot increases the reachable workspace, especially in front of the users leg space, e.g. to reach the ground. The robot arm is equipped with the DLR-Clash hand which provides joint impedance control with intrinsic compliance capabilities^58^. The system can be commanded by any continuous 3D input signal, such as 3-DoF joysticks or 3-DoF interfaces based on biosignals. To use an sEMG-based interface, remaining voluntary muscle activation must be measurable along the extremities of the user.

**Fig. 4:**
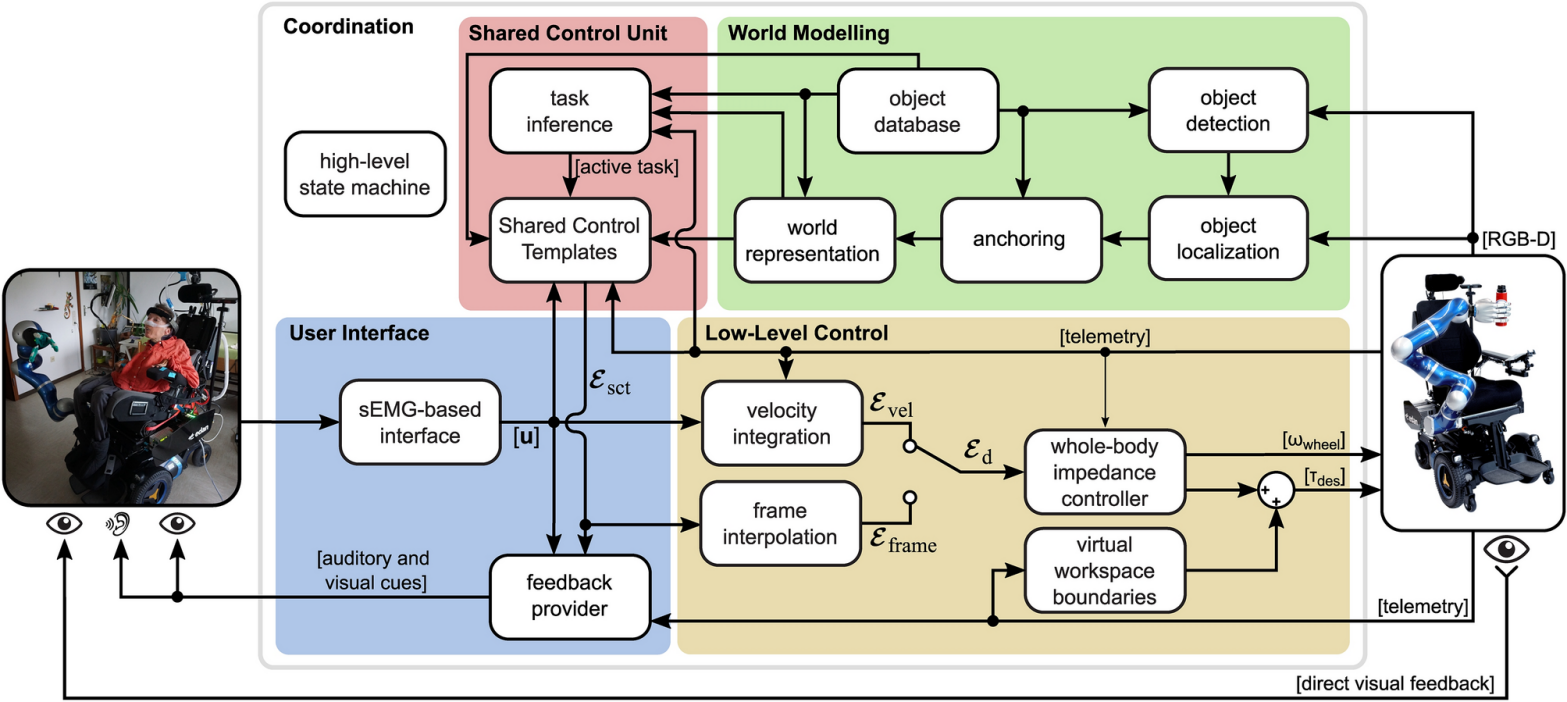
System diagram, with software infrastructure and control modes. The modular software framework of EDAN can be divided in four parts: the user interface, the shared control unit, the world modeling, and the low-level control. The overall workflow is managed by a high-level state machine. While the user gives input to control the robot via the bioelectrical-signal-interface, the system provides visual feedback on a tablet screen. In direct control, the desired EE position and orientation for the whole-body controller ($\varvec{\mathcal {E}}_\textrm{d}$) is determined through velocity integration ($\varvec{\mathcal {E}}_\textrm{vel}$). In shared control, it is determined through frame interpolation ($\varvec{\mathcal {E}}_\textrm{frame}$) based on the desired EE pose computed by the Shared Control Templates ($\varvec{\mathcal {E}}_\textrm{sct}$). The world model holds an instantiation of the scene including known objects, which serves as a basis for shared control.

Additional magnetic encoders are integrated into the front wheels of EDAN, enabling precise measurement of the wheelchair position and odometry calculation. An RGB-D camera observes the scenery in front of EDAN and detects known objects within the robots surrounding. All needed information about the system, such as detected objects, or the used control scheme, was presented via the GUI, displayed on a tablet. The required software modules for low- and high-level control as well the perception processes were all calculated on board. An overview of the individual software modules is provided in Fig. 4 as well as in the following.

#### User interface

The sEMG-based interface converts muscular activity measured with surface electromyography into continuous 3D control signals. Spots on the dominant arm of the participants with voluntarily muscle activation were identified for up to eight sensors. The spots were identified during their first session and used for all following sessions (except for two sensors of P-C, which were repositioned in the 4th session). The training procedure was completed at the beginning of each session. sEMG signals were preprocessed using the time domain feature set^59^ and mapped to 3D commands with Gaussian process (GP) regression. For details see SM 2.1.1. Three independent GPs were calculated, one for each DoF: u1,u2,u3 (corresponding to motions in the x, y and z direction in translationnal control for example), allowing combined activation, i.e. to evoke diagonal motions. A binary trigger signal was additionally decoded through a Linear Discriminant Analysis (LDA) classifier and used to click on the GUI (shown on the tablet) or during shared control to start a task when no concrete target frame was defined, e. g. for releasing a grasped object on a table.

The hybrid interface (used by P-C) combines the signal of a 2-DoF joystick for the x- and y-directions with the z-component and binary signal decoded from muscular activity. To disambiguate muscular activity related to joystick movement from that related to the needed z-component, the training procedure was similar to that of the 3D sEMG-interface: three individual GPs were trained, x- and y-directions were labeled with zero.

Besides the sEMG-based interface a head switch is used to manage the switching between the different devices *robotic arm*, *wheelchair*, and *tablet*. If the tablet is selected, the user can change the control scheme via the sEMG-based interface.

#### World modeling

The system’s world model includes the detection of objects in the environment and handles geometric information required to manipulate them. From an object database^60^ and an RGB-D camera, a 6D pose of known objects is estimated based on a two-stage perception pipeline: object detection^61^ for class categorization followed by object localization^62^. An anchoring algorithm tracks and filter those estimations to assign symbolic tags to specific instances. Anchored objects are then instantiated in a world state representation^60^, which provides information about available skills and objects attributes such as poses and grasp frames to the shared control unit.

#### Shared control unit

*Task inference*: The world model instantiated with objects keeps track of symbolic information, such as whether the manipulator is holding an object or whether a bottle contains water. The task inference module identifies which tasks are currently possible, based on the state of the objects in the world model and the symbolic task preconditions, represented in the Planning Domain Definition Language (PDDL)^63^. Based on the EE distance to the target of the task and the task constraints, the inference additionally keeps track of how likely each task is to be the user’s current desired task. This information is continuously displayed on the graphical user interface. Based on predefined thresholds, the task inference activates shared control for a task automatically. Alternatively, the user can select a task from a list of currently possible tasks in the tablet GUI. See also Supplementary Materials SM 2.5 for details.

*Shared control templates*: SCTs provide assistance for the user by guiding and constraining the EE movement during task execution, while the user is in control of the robot at any time^33^. There is no robot motion applied if the user gives no input. An SCT is represented as a finite state machine, e.g. ‘approach cup’–‘tilt bottle’–‘pour’ in a pouring task. Transitions between states are triggered by distance metrics (e.g. the distance to the cup), contact forces or EE sensors values. In each state, input mappings and active constraints provide the relevant support. An input mapping maps user input commands to velocities applied on the desired EE frame. In the ‘approach cup’ state for instance, the 3D input command maps directly to 3-DoF translational EE movements, as in direct control. In the ‘pour’ state, two DoFs of the user command are mapped to a rotation of the grasped bottle around its tip, while the last component is used for vertical translation to adjust the pouring height.

An active constraint restricts the task space by projecting the current EE pose to a constrained pose^64^. For example, enforcing a maximum tilting angle during pouring, or constraining a reaching motion to guide it towards a specific grasp frame for a door handle. The procedure to add new SCTs, for example for a new object, is straightforward: once the object has been added to the object database and can be detected by the perception system, various skills can be created by defining a task specific state machine with according input mappings and active constraints. See also SM 2.6.

#### Low-level control

The 8-DoF robotic manipulator is commanded in torque control, utilizing a Cartesian Impedance controller. The remaining 2-DoF nullspace allows for definition of an additional (secondary) task, namely the Cartesian position of the elbow joint, which is important for tasks in confined spaces, like passing through a door (see SM 2.3.1).

Two different interpolation strategies are applied, depending on the control scheme, see Fig. 4. During direct control, incremental interpolation is used to treat the user input as a velocity command applied at the EE. During shared control, a desired pose is first calculated from the shared control module at a rate of 30 Hz. A recommandable frame interpolator then calculates a smooth trajectory that complies with defined velocity limits in translation and rotation. See also SM 2.3.4 and 2.3.5.

To ensure user safety, workspace restrictions prevent direct contact between manipulator and the user. These so called virtual walls generate repulsive forces that are converted into joint torques that are added to the desired torques of the Cartesian Impedance controller, see also SM 2.3.3.

*Whole-body impedance control:* The whole-body controller computes commands that couple arm and wheelchair motions. The motion of the mobile platform is generated through the forces resulting from a virtual spring within the whole-body control scheme, see Iskandar et al.^36^ and SM 2.3.2. These forces are transformed into velocity-commands via an admittance interface, and the desired motion is realized using a proportional-integral (PI) velocity controller. During shared control, whole-body control increases the kinematic reachability of the manipulator by moving the wheelchair based on task specific workspace boundaries. In our system, whole-body control is activated automatically in tasks where reachability of the arm may not be sufficient.

## Supplementary Information

Supplementary Information 1.

Supplementary Information 2.

Supplementary Information 3.

Supplementary Information 4.

Supplementary Information 5.

Supplementary Information 6.

## Data Availability

The datasets generated and analyzed during the current study are available from the corresponding author on reasonable request.
