# Supplementary material for: An assistive robot that enables people with amyotrophia to perform sequences of everyday activities
Source: Sci Rep. 2025 Mar 11;15:8426. doi: 10.1038/s41598-025-89405-2 (PMC11897195; doi:10.1038/s41598-025-89405-2)
Supplement: Supplementary file 1 — Supplementary Information 1. [file 41598_2025_89405_MOESM1_ESM.pdf]

Supplementary Materials

1 Pilot study

1.1 Adjustment of the EDAN system

The EDAN system had to be adjusted to the individual needs of the participants. Figure 1 depicts all three participants sitting in the wheelchair. P-A and P-C were able to sit in the wheelchair with the existing seat cushions. In the case of P-B, a special seat shell had to be inserted into the EDAN system.

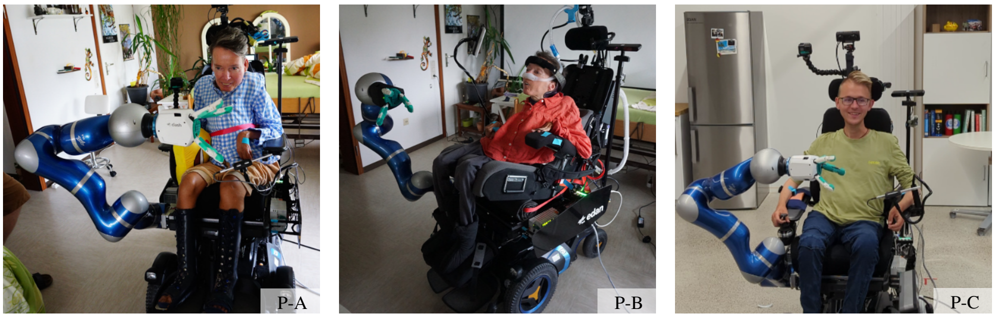

Fig. 1: **Participants sitting in the individually adjusted EDAN system with an electromyography-based interface.** While P-A and P-C could use the existing cushions, for P-B a special seating shell was used for her to be able to sit in the system.

1.2 Details about experimental procedure

In total 6 sessions were executed with P-A and P-B and 5 with P-C. To keep the time in which they sat in the wheelchair short and in order to not create too much cognitive strain, a session duration was limited to a maximum of 3 hours. Due to limited sitting capabilities in the case of P-A and P-B, the sEMG-sensor placement and training of the interface were done while participants were lying in bed next to the system. Afterwards, the participants were transferred into the wheelchair by a qualified caretaker. These preparations (including sensor placement, training of the interface, transfer to the wheelchair) had to be performed at the beginning of each session. This resulted in an effective session time of about 1.5 hours per session.
For P-A, the individual sessions were organized as shown in Table 1.

**Session 1**	Adjustment of the wheelchair and electrode placement.
**Session 2**	Familiarization with the interface and the system, lying next to the system.
**Session 3**	Familiarization with the interface and the system, lying next to the system.
**Session 4**	Familiarization with the system, while sitting in the wheelchair and performing tasks of daily living.
**Session 5**	Performing tasks of daily living using the whole system.
**Session 6**	Performing tasks of daily living using the whole system.

Table 1: Individual sessions of **P-A**

**Session 1**	Adjustment of the wheelchair and electrode placement.
**Session 2**	Familiarization with the interface and the system, lying next to the system.
**Session 3**	Familiarization with the interface and the system, lying next to the system.
**Session 4**	Performing tasks of daily living using the whole system, lying next to it.
**Session 5**	Familiarization with the system, while sitting in the wheelchair.

Table 2: Individual sessions of **P-B**

The initial plan for P-B was to follow the same schedule as P-A. However, the unexpected unavailability of qualified caregivers precluded a transfer into the wheelchair, resulting in only one out of the five sessions with P-B sitting in EDAN. Thus, a total of only five sessions, including one session sitting in EDAN, was possible for P-B. The individual sessions of P-B were organized as shown in Table 2. The individual sessions of P-C were organized as shown in Table 3.

**Session 1**	Adjustment of the wheelchair and electrode placement.
**Session 2**	Familiarization with the interface and the system, sitting in the wheelchair.
**Session 3**	Familiarization with the interface and the system, sitting in the wheelchair.
**Session 4**	Performing a Fitts' Law Test as well as tasks of daily living using the whole system.
**Session 5**	Performing tasks of daily living using the whole system.

Table 3: Individual sessions of **P-C**

1.3 Additional results

To evaluate the overall performance of the hybrid interface and to detect directional differences, a 3D Fitts' Law Test with 32 goals was conducted in P-C's fourth session. Given that motions in the x-y-plane were commanded from the joystick while vertical z-motions originated from the sEMG signals, we separated the goal positions in 2 equally sized bins split at the median of z-motion required to reach the target. Goals with higher z-component resulted in an Index of Performance (IP) – also called throughput – of 0.69, while goals having lower Z-component lead to an IP of 0.84. Figure 2 shows the results of a 3D Fitts' Law Test done by participant P-C. An illustration is given showing the desired end effector (EE) motion as well as the actual EE movement done by P-C. The additional table shows the respective motion time for the four different distances and index of difficulty (ID). The index of difficulty and index of performance (IP) of the Fitts' Law Test are defined as in [1].

2 The EDAN system

EDAN uses Shared Control Templates (SCT) to support during a range of tasks of daily living. At the time of the experiments, the system could support with the following tasks: grasping, placing, pouring and drinking with a bottle, grasping, placing and drinking with a mug, opening and closing a drawer, opening a fridge, and opening and driving through a door.

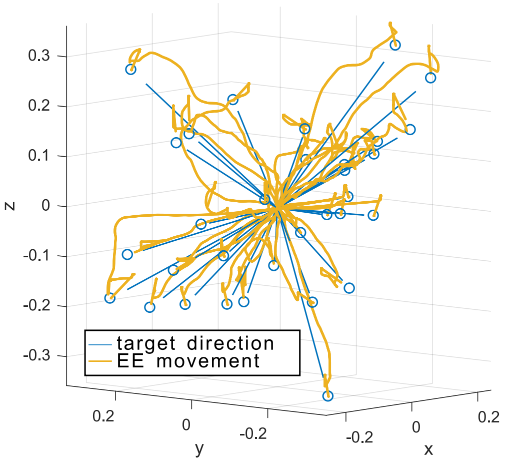

Movements of the EE during the Fitts' Law test for 32 goals, shown as blue circles.

D [m]	ID	MT [sec]
0.16	1.87	2.52
0.24	2.32	2.88
0.32	2.66	3.96
0.4	2.94	4.57

The motion time MT shows the average time to reach a target with a specific distance D and the calculated index of difficulty ID.

Fig. 2: **Results for the Fitts´ Law Test in 3D done by P-C using the hybrid interface.** Left: EE movements; Right: motion time per ID.

The hardware design of the system includes a commercially available wheelchair equipped with an 8-axis version of the DLR Light Weight Robot III and a DLR-Clash hand. The wheelchair is controlled via the proprietary R-NET interface, through which forward-backward and rotational velocity commands can be sent with an analog signal.

The system is equipped with a Linux-based real-time computer (Intel I7 4-Cores), which runs the low-level control software. Another Linux-based computer (Intel I7 8-Cores) is used for the high-level control software. Object detection and localization is performed on a Nvidia Jetson TX2 embedded GPU in combination with an ASUS Xtion RGB-D Camera. Processes for the DLR-Clash hand were computed on an additional Intel-based embedded computer. An overview of the individual software modules is provided in the following.

2.1 User interfaces

2.1.1 sEMG-based Interface

Both participants P-A and P-B have spinal muscular atrophy in an advanced state, which severely inhibits their motion capabilities. SMA leads to the death of motor neurons in the spinal cord resulting in a progressive amyotrophia. For P-A, the remaining movement of the thumb and index finger allows the use of a personalized micro 2-Degrees of Freedom (DoF) joystick. However, the residual muscular activity is not sufficient for vertical motion, hence not allowing the use of a 3-DoF joystick. Participant P-B lost most voluntary movement in her fingers (except for minor motions of the index finger) which prevents the use of a joystick at all. She uses a 2-DoF chin joystick to move her wheelchair in daily life. This interface does not provide continuous 3D control. Both participants used the 3D sEMG-based interface with an additional sEMG-based trigger to continuously control the motion of the robot arm in task space.

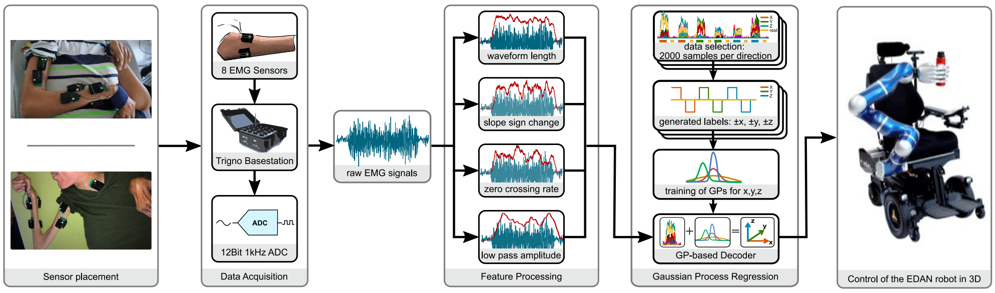

Fig. 3: **Schematic overview of the signal processing pipeline for the sEMG-based interface.** *Sensor placement*: participants P-A and P-B with their individual sensor placements. *Data acquisition*: process of converting the sEMG-signals to digital signals. *Feature Processing*: using a sliding window of 150 samples to calculate the four time-domain features. *Gaussian Process (GP) Regression*: training procedure to get a GP-based decoder using three labeled training sets. *EDAN*: the robotic system to be controlled.

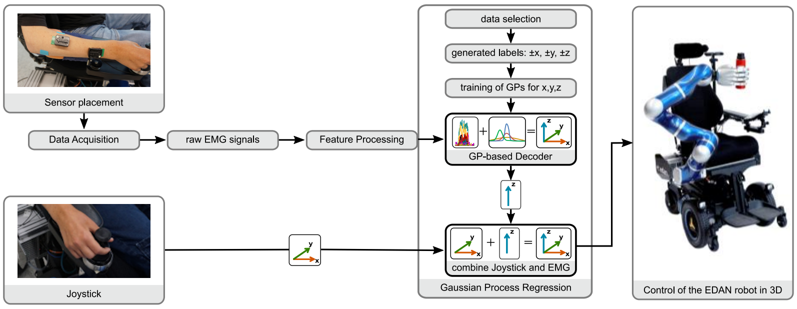

Fig. 4: **Schematic overview of the hybrid interface.** The training procedure is identical to the sEMG-based interface. For commanding, the x-,y-components originate from the Joystick, while the z-component is decoded from sEMG-signals. These combined signals are then applied to 3D robot control.

Participant P-C is affected by muscular dystrophy resulting in very limited arm and hand function. However, P-C has enough motor capability left to use a regular 2-DoF joystick to control a motorized wheelchair in everyday life. To make use of his remaining hand function, P-C used a hybrid interface, consisting of a 2-DoF joystick with additional control signals extracted from sEMG for the third DoF and the binary trigger signal.

Sensor placement

Up to eight Trigno® wireless electromyography sensors from the company Delsys were attached to the arm of the participants using a double-sided adhesive medical tape. Electrodes positions along the arm were individually and manually selected to allow for the recording of the participants remaining voluntary muscle contractions. In the case of P-A and P-C, the main sensor used for the trigger signal was placed on the skin of the left arm, which optimized the precision of the sEMG-based trigger. Due to progressed amyotrophy these places were very selective and positions were marked for following sessions. In the case of P-C, the activation strategy changed during session 3, which led to a changed sensor placement in session 4 and 5. Individual sensor placement can be found in Table 4.

Sensor #	P-A	P-B	P-C
1	Flexor digiti minimi	Thenar	Extensor digitorum
2	Thenar	Flexor digiti minimi	Triceps
3	Biceps brachii	Flexor carpi radialis	Extensor digitorum (left)
4	Extensor indicis	Extensor carpi ulnaris	Brachioradialis
5	Flexor digitorum	Biceps brachii	Extensor carpi ulnaris
6	Trapezius	Triceps brachii	Flexor carpi radialis
7	Thenar (left)	Deltoid	—
8	Biceps brachii	Sternocleidomastoid	—

Table 4: **Final sEMG-sensor placement for all participants.** The listed sensor locations refer to the muscles of the right arm which are anatomically closest to the location of the sensor. Marked with '(left)' refers to sensors placed on the left arm. Due to severe muscular atrophy actual muscles that have been recorded may differ from this list.

Data processing

Acquired raw sEMG-signals were locally amplified within the sensors and transferred to the Delsys Trigno® base station. An analog-to-digital converter of the company Beckhoff was used to digitize the ±5 V analog signal from the base station into a 12-bit signal at a rate of 1 kHz. A linux real-time computer received the data via EtherCAT, where the signal was further processed at 1 kHz. In a first processing step, a bias is subtracted from the sEMG data-stream in order to remove the DC component of the sEMG signal. The data of the sensors is prepossessed using the time-domain features introduced by Hudgins et al. [2], including sEMG-amplitude, slope sign-change, zero-crossing-rate and waveform-length.

All features were calculated online with a sliding window of 150 samples on each sEMG-channel. A schematic overview of the signal processing can be found in Figure 3.

Training procedure

Training data was gathered before calculating a prediction model. Muscle signals are measured for 5 seconds while the arm is at rest (without voluntary muscle activity). This data is used to calculate the baseline noise, which is subtracted from the sEMG data-stream. Furthermore, this recording allowed identification of an individual activity threshold to distinguish between rest and voluntary activation, which was used for the remaining training procedure. The calculation of the threshold was based on a binary search. For details see [3].

In total, 3 DoFs i.e. six directions were decoded with the interface. Therefore, participants were asked to provide muscle activity which they intended to associate with motions along the cardinal axes: upward, downward, leftward, rightward, forward, and backward. One training dataset included 2000 samples of activity per direction as well as 1000 samples of pause (rest state) in between. This data was labeled with the respective direction or as “not active”. The desired direction was announced by a computer voice. Muscular activity was recorded as soon as the activity threshold was exceeded and the robot gave feedback by moving in the respective direction. The recording of each direction took approximately 2 seconds and a full training cycle was performed in less than 30 seconds. Four training cycles were recorded at the beginning of each session out of which the last three were used for training the models. Additionally, one dataset was recorded to train the classifier for the sEMG-based trigger. For this data set, the participants were asked to give 5 impulses of muscular activity associated with the trigger, alternating with pauses in muscle activity.

Decoding with Gaussian process regression

Gaussian process regression was used in order to decode the continuous velocity commands $\boldsymbol{u}(t)$ for robot control. The pyGP library [4] was utilized, which is based on the implementation of [5]. Three independent GPs were calculated, i.e. one GP for $\pm$x, one for $\pm$y, and one for $\pm$z, respectively. With individual models, a command can be a linear combination of the estimated command along each axis, i. e. motions in any direction are possible, not just along the x-,y-,or z-directions. The activity threshold allowed the participants to stop robot motion, as the GP output was only applied when the threshold was exceeded, otherwise the robot position was kept static.

Hybrid interface

The initialization of the hybrid interface was conducted similarly to that of the full 3D interface: all three DoFs were recorded and trained with the predictive model, whereas just one of them (the z-component) was used as input to the system, see Figure 4. Nevertheless, training was executed in all directions (while giving usual joystick commands in x- and y-direction), to record muscle signals which occurred during these directions and label them as zero for the z-component.

2.1.2 Feedback Provider: Tablet

A tablet mounted on the wheelchair displays a Graphical User Interface (GUI) with all the relevant information to the user. Figure 5 shows the GUI displayed to the user and its different components (A-F).

By operating the head-switch, the user can cycle through the activation of the different devices, including the tablet. By selecting the tablet, its view changes and the user can choose between the different control schemes like shared control or direct control. Furthermore, a list of all possible tasks based on the current world model status is provided. The user can start one of these by selecting it, even if it is not the most likely task according to the task inference. The sEMG-based interface used to control the robot serves in the same manner to control the tablet: the x- and y-components of the control command are used to select items, while the sEMG-based trigger serves as a click to select the highlighted item, e. g. control schemes or tasks.

2.2 Software Coordination

EDAN's software is coordinated by an event-based finite state machine. There are various types of system states that need to be coordinated: which device the user wants to control ('tablet GUI', 'arm', 'wheelchair', or 'nothing'), the interpolator used (frame, velocity), the control mode of the wheelchair (with or without whole-body control, only translations or rotations), the active task, etc. The various combinations of those control modes form the high-level states the EDAN robot can be in. The user can change theses states by selecting a task on the tablet or completing the execution of a task like releasing an object. Processes can as well change those states, e. g. the task inference starting shared control support for *pick* using the frame interpolator once close enough to a pick-able object.

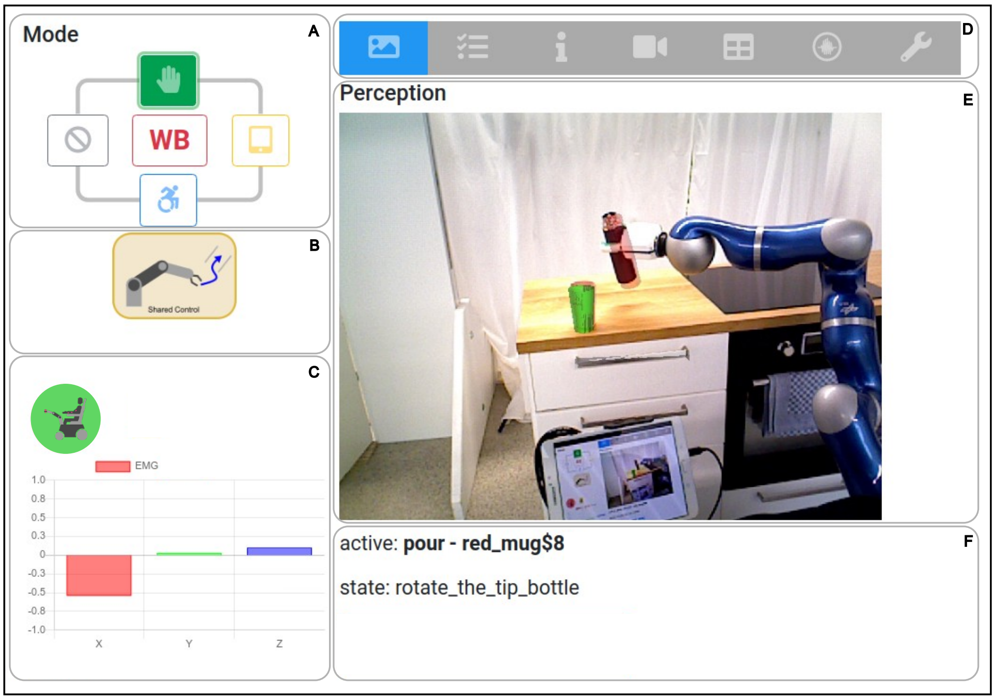

Fig. 5: **The GUI, which was displayed on a tablet. A:** Device controlled by the user, cycled through by operating the head-switch: 'robot control', 'tablet', 'wheelchair control', or 'nothing' (user commands have no effect to the system). Furthermore, [WB] is highlighted if the whole-body control scheme is active. **B:** Active control scheme (direct or shared). **C:** Decoded commands. The green circle provides information if the activity threshold is exceeded to allow a control input. **D:** Additional tabs used to display the list of control schemes, the list of tasks, and expert information not needed by the participants. **E:** World model visualization; shown is the RGB-camera stream augmented with the localized objects instantiated in the world model. Different colors highlight different states of the objects, like green which highlights the target object of the current task. **F:** Information regarding the current task and states; shown is the active task as well as the current state of the task.

2.3 Real-Time Processes

2.3.1 Whole-Body Cartesian Impedance Control

Assistive robotic systems in daily support scenarios are exposed to constant physical interaction with the environment. Thus, an exchange of interaction forces between the robotic system and the surrounding environment is expected. Accordingly, a compliant behavior at the robot EE is desirable, which can be achieved by active control. The EDAN system features a reactive whole-body control that allows coordination between the mobile base (the wheelchair) and the robotic arm. This facilitates the execution of tasks beyond the kinematic reachability of the manipulator, e.g. opening a door. To ensure passive physical interaction with the environment we command the joint torques to realize Cartesian impedance behavior as

$$\boldsymbol{\tau} = \boldsymbol{\tau}_{\text{imp}} + \boldsymbol{\tau}_{\text{null}} + \boldsymbol{\tau}_g(\boldsymbol{q}), \tag{1}$$

where $\boldsymbol{\tau}_{\text{imp}}$ is the generated torque to actively realize the Cartesian impedance at the EE, the term $\boldsymbol{\tau}_{\text{null}}$ achieves a null space secondary task, and $\boldsymbol{\tau}_g(\boldsymbol{q})$ stands for the gravity compensation component. The Cartesian impedance control action can be formulated as

$$
\begin{aligned}
\boldsymbol{\tau}_{\text{imp}} =& - \boldsymbol{J}^T(\boldsymbol{q})(\boldsymbol{K}_x\tilde{\boldsymbol{x}}(\boldsymbol{q}) + \boldsymbol{D}_x\dot{\boldsymbol{x}}) \\
\tilde{\boldsymbol{x}}(\boldsymbol{q}) =& \boldsymbol{f}(\boldsymbol{q}) - \boldsymbol{x}_{\text{d}} \\
\dot{\boldsymbol{x}}(\boldsymbol{q}) =& \boldsymbol{J}(\boldsymbol{q})\dot{\boldsymbol{q}}.
\end{aligned}
$$

The mapping $\boldsymbol{f}(\boldsymbol{q}) : \Re^n \rightarrow \Re^6$ encodes the forward kinematics while $\boldsymbol{x}_{\text{d}} \in \Re^6$ denotes the desired task-space position and orientation. The Cartesian stiffness and damping are represented by the positive definite matrices $\boldsymbol{K}_x, \boldsymbol{D}_x \in \Re^{6\times6}$, respectively. The controller relies on the low-level torque control loops to realize the desired impedance. However, the torque dynamics are assumed to be much faster such that one can obtain the desired impedance behavior [6, 7].

In our framework of hierarchical whole-body control, the elbow position is regulated to obtain a specific position in the nullspace of the arm. This desired elbow position is accessible from the higher-level software and can thereby be defined for individual states of an SCT-skill. This is used to keep the elbow in an outward position when manipulating objects, or to achieve a more compact configuration of the system (in term of overall system width), for instance when passing through a door. The control action $\boldsymbol{\tau}_{\text{null}}$ is realized in the null space of the main EE task as

$$\boldsymbol{\tau}_{\text{null}} = -\boldsymbol{N}\boldsymbol{J}_{\text{e}}(\boldsymbol{q})^T\boldsymbol{f}_{\text{e}}.$$

Here, the task-space null space projector is denoted by $\boldsymbol{N} \in \Re^{n\times n}$, and $\boldsymbol{J}_{\text{e}}(\boldsymbol{q}) \in \Re^{6\times n}$ and $\boldsymbol{f}_{\text{e}} \in \Re^6$ are the elbow Jacobian matrix and control wrench, respectively. Similar to the main EE task mentioned previously, the robot elbow control action is calculated to realize a desired Cartesian stiffness and damping such that $\boldsymbol{f}_{\text{e}} = (\boldsymbol{K}_e\tilde{\boldsymbol{x}}_e + \boldsymbol{D}_e\dot{\boldsymbol{x}}_e)$ at the elbow location $\boldsymbol{x}_e$.

2.3.2 Platform Motion Control

The actuation signals that are applied to the mobile base (wheelchair) are calculated by introducing an additional virtual spring of which the deflection depends on the EE boundaries. As shown in Figure 6 these boundaries are defined in a line or arc w.r.t the mobile base and can be parameterized by $[x_{\text{min}}; r_x]$, $[y_{\text{min}}; y_{\text{max}}]$. Two non-linear functions $h_1(x_\mathcal{E}, y_\mathcal{E})$ and $h_2(y_\mathcal{E})$ are defined to create the activation area of the wheelchair motion as

$$h_1(x_\mathcal{E}, y_\mathcal{E}) = \begin{cases} \sqrt{x_\mathcal{E}^2 + y_\mathcal{E}^2} - r_x & \text{, if } \sqrt{x_\mathcal{E}^2 + y_\mathcal{E}^2} \geq r_x \\ x_\mathcal{E} - x_{\text{min}} & \text{, if } x_\mathcal{E} < x_{\text{min}} \\ 0 & \text{, else} \end{cases}$$

and

$$h_2(y_\mathcal{E}) = \begin{cases} y_\mathcal{E} - y_{\text{max}} & \text{, if } y_\mathcal{E} \geq y_{\text{max}} \\ y_\mathcal{E} - y_{\text{min}} & \text{, if } y_\mathcal{E} < y_{\text{min}} \\ 0 & \text{, else} \end{cases}$$

where $x_\mathcal{E}$ and $y_\mathcal{E}$ are two components from the EE position w.r.t. the manipulator base frame $\mathcal{W}$. As a result, the virtual motion activation boundaries move with the mobile platform,

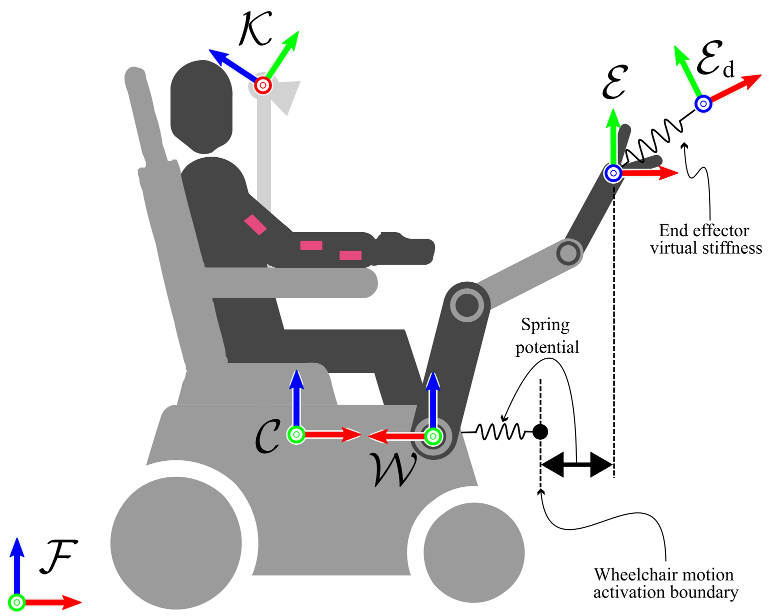

Fig. 6: **Schematic illustration of the whole-body Cartesian impedance control.** The coordinate frames used by the system are highlighted. $\mathcal{K}$ is the camera frame, $\mathcal{F}$ a static frame in the environment, $\mathcal{C}$ the center of the wheelchair, $\mathcal{W}$ the base of the robotic manipulator, $\mathcal{E}$ the EE frame, $\mathcal{E}_\mathrm{d}$ the desired EE frame.

such that a behavior transparent to the user is achieved. The commanded platform forces are obtained via a potential function as

$$\boldsymbol{\tau}_b = \boldsymbol{K}_\mathrm{WB} \begin{bmatrix} h_1(x_\mathcal{E}, y_\mathcal{E}) & 0 \\ 0 & h_2(y_\mathcal{E}) \end{bmatrix}$$

where $\boldsymbol{K}_\mathrm{WB} \in \Re^{2\times 2}$ is a diagonal stiffness matrix. Once the boundaries are exceeded a potential starts to build from the wheelchairs virtual spring $\boldsymbol{K}_\mathrm{WB}$ that results in commanded forces to the platform as visualized in Figure 6. The potential is generated by the output of the function $h_1(x_\mathcal{E}, y_\mathcal{E})$ in the forward and backward directions and $h_2(y_\mathcal{E})$ is applied to the rotational direction. During whole-body control tasks the user can give Cartesian velocity commands to the EE without explicitly considering the wheelchair motion. Moreover, the parameters of the platform motion generation functions can be adapted online based on the state of a task to ensure reachability of the arm. Additionally, $\boldsymbol{\tau}_b$ can be combined from different sources, for example with an external force observer to specify interaction safety thresholds [8] or avoid collisions with the environment.

The commanded platform forces are transformed to the required velocities $\boldsymbol{\eta}_\mathrm{d}$ through an admittance interface as

$$\boldsymbol{M}_\mathrm{adm}\dot{\boldsymbol{\eta}}_\mathrm{d} + \boldsymbol{D}_\mathrm{adm}\boldsymbol{\eta}_\mathrm{d} = \boldsymbol{\tau}_b + \boldsymbol{\tau}_b^\mathrm{ext}.$$

The desired mobile base behavior can be specified using the parameters $\boldsymbol{M}_\mathrm{adm}$, $\boldsymbol{D}_\mathrm{adm}$ which are the virtual platform inertia and damping of the admittance interface, respectively. The

generalized external forces $\boldsymbol{\tau}_b^{\text{ext}}$ are obtained using the external joint torques to realize whole-body impedance causality. Furthermore, a lower-level proportional-integral controller is used to realize the desired velocity of the wheelchair. The actual base velocity is obtained from the wheelchair odometry model as a function of wheel velocities.

2.3.3 Virtual Workspace Boundaries

Based on functional requirements the user has to be located in the workspace of the robot, e.g. in order to bring a filled drinking container to their mouth. For a safe application, we generate a virtual environment to restrict the workspace of the manipulator to prevent direct contact between user and manipulator.

This virtual environment generates virtual obstacle forces that are converted to joint torques and can be added to the control scheme. We therefore extend Equation 1 with $\boldsymbol{\tau}_{\text{d,VE}}$, which is the desired joint torque resulting from the virtual environment:

$$\boldsymbol{\tau} = \boldsymbol{\tau}_{\text{imp}} + \boldsymbol{\tau}_{\text{null}} + \boldsymbol{\tau}_{\text{d,VE}} + \boldsymbol{\tau}_g(\boldsymbol{q}).$$

The virtual environment consists of planes, spheres and cylinders which mark zones the robot shall not enter, specifically adjusted for each user. In case of penetration of the workspace boundary, the Cartesian EE velocity $\dot{\boldsymbol{x}}$, the current EE pose $\boldsymbol{x}$ and the pose resulting from the projection of $\boldsymbol{x}$ on the surface of the virtual environment $\boldsymbol{x}_{\text{d,VE}}$ can be used to calculate a workspace boundary wrench $\boldsymbol{f}_{\text{V}i}$ as

$$\boldsymbol{f}_{\text{V}i} = K_{\text{V}i}(\boldsymbol{x} - \boldsymbol{x}_{\text{d,VE}}) + D_{\text{V}i}\dot{\boldsymbol{x}}_i.$$

Given N workspace boundary wrenches, the resulting torque $\boldsymbol{\tau}_{\text{d,VE}}$ generated by the virtual environment for the workspace limits is defined as

$$\boldsymbol{\tau}_{\text{d,VE}} = \sum_{i=1}^{N} \boldsymbol{J}^T \boldsymbol{f}_{\text{V}i}.$$

Additional workspace boundaries for other parts of the robot structure are defined by using the forward kinematics and the corresponding Jacobian matrix of the respective joint of the manipulator.

2.3.4 Velocity Integration

When using the direct control scheme, the user input $\boldsymbol{u}$ is treated as a velocity command applied at the EE. Dependent on the current subset of DoFs to be controlled, this velocity command is either integrated to the translational or rotational component of the EE pose. In case of grasp selection, the user command is treated as a joint velocity command that is applied to the joint configuration of the robotic hand. The user input $\boldsymbol{u} \in \Re^3$, $\boldsymbol{u} \in [-1, 1]^3$ is a unit-less vector which is multiplied with the defined maximum control velocities k_{trans}, k_{rot} and k_{grasp} for translational, rotational or grasping motion, respectively.

$$\dot{\boldsymbol{x}}_{\text{d}} = k\boldsymbol{u},$$

$$\text{with } k \in [k_{\text{trans}}, k_{\text{rot}}, k_{\text{grasp}}]$$

In order to also account for the workspace limits at the desired position and velocity level, we define the desired position $\boldsymbol{x}_{\mathrm{d}}$ as

$$\boldsymbol{x}_{\mathrm{d}} = \int_0^t [\dot{\boldsymbol{x}}_{\mathrm{d}} - \boldsymbol{c}(\boldsymbol{f}_{\mathrm{V}}, \dot{\boldsymbol{x}}_{\mathrm{d}})].$$

The state-dependent vector $\boldsymbol{c}(\boldsymbol{f}_{\mathrm{V}}, \dot{\boldsymbol{x}}_{\mathrm{d}})$ accounts for hitting workspace limits by stopping translational integration of the decoded velocity signal if such a boundary is hit and the velocity direction points towards the wall:

$$\boldsymbol{c}(\boldsymbol{f}_{\mathrm{V}}, \dot{\boldsymbol{x}}_{\mathrm{d}}) = \begin{cases} \frac{-\boldsymbol{f}_{\mathrm{V}} \cdot \dot{\boldsymbol{x}}_{\mathrm{d}}}{\|\boldsymbol{f}_{\mathrm{V}}\| \|\dot{\boldsymbol{x}}_{\mathrm{d}}\|} \frac{\boldsymbol{f}_{\mathrm{V}}}{\|\boldsymbol{f}_{\mathrm{V}}\|} & \text{, if } -\boldsymbol{f}_{\mathrm{V}} \cdot \dot{\boldsymbol{x}}_{\mathrm{d}} > 0 \\ 0 & \text{, else} \end{cases}$$

where $\boldsymbol{f}_{\mathrm{V}} = \sum \boldsymbol{f}_{\mathrm{V}i}$ is the workspace boundary wrench generated by the virtual environment.

2.3.5 Frame Interpolation

When using the direct control mode, continuity of the robot's desired pose is ensured from the integration process. This property is not given, when using our shared control approach. From a Shared Control Template, a desired pose $\boldsymbol{X}_{\mathrm{SCT}} \in SE(3)$ for the EE is calculated online at a rate of 30Hz, which can not be immediately applied to the Cartesian impedance controller running at a rate of 1kHz. Instead, we utilize a frame interpolation module that follows two objectives: one, continuously follow a target pose that is provided at a comparably low rate; two, ensure limits in the translational and rotational velocity of the resulting trajectory.

To achieve this, the frame interpolator calculates a linear interpolation towards the current SCT desired frame $\boldsymbol{X}_{\mathrm{SCT}}$. This is trivial to achieve for the translational component. For the rotational component, spherical linear interpolation in the quaternion representation [9] is used. Linear interpolation allows for a straightforward integration of a velocity limit through adjustment of the interpolation time T_m. In order to prevent the robot from moving faster than the source signal, we define the final interpolation time $T_q = \max(\mathrm{T_m}, \mathrm{T_s})$ where $T_s = 1/30\mathrm{Hz}$ is the sample time of the shared control module.

This piece-wise linear interpolation results in discontinuities in the velocities of the resulting trajectory. In order to create a smooth trajectory, which can be used as input to the Cartesian impedance controller, a second order filter is applied. More details on the frame interpolator approach is available in [10].

2.4 World Modeling

The world modeling pipeline is summarized in Figure 7 and involves multiple components including an Object Database storing object specific knowledge, an Object Detector to find the relevant object in the image and for class categorization, and an Object Localization for precise 6D pose estimation. After this, an Anchoring algorithm assigns symbolic tags to specific instances, which are instantiated in a World State Representation (WSR) process. More information of these modules is provided in the following subsections.

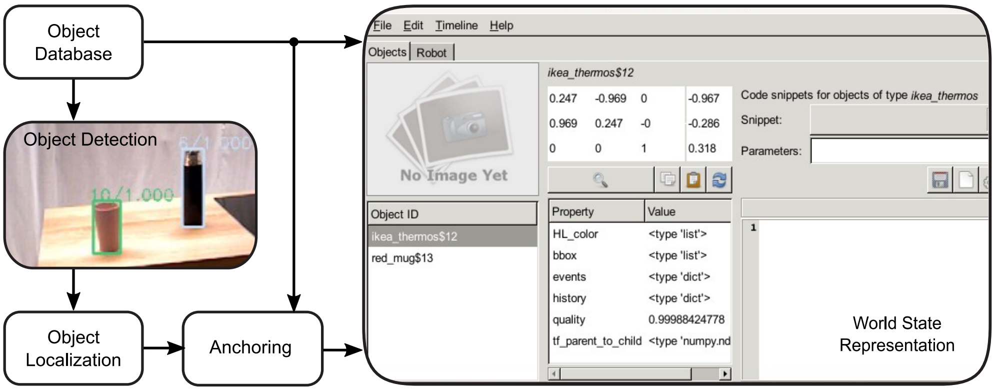

Fig. 7: **Components of EDAN's world modeling module.** Right: shown the Object Data Base (ODB) [11].

2.4.1 Object Database

The Object DataBase (ODB) was first introduced in Leidner et. al. [11]. It stores any object information that is available to the robot *a priori*, including attributes (like color, mass or length), frames (like the tip of a bottle expressed in the origin frame of this bottle), robot properties (like grasp configurations), SCTs (stored as *yaml* files) and 3D models.

An important property of the ODB is its object oriented paradigm: physical objects (like EDAN's red_mug) are derived from abstract classes (like _mug, which itself derives from _container). This enables a *polymorphism* feature. As a concrete example, the same SCT (releasing a cylindrical container) can be inherited by different objects (a red_mug and a lab_bottle) and instantiated with different properties and frames [11].

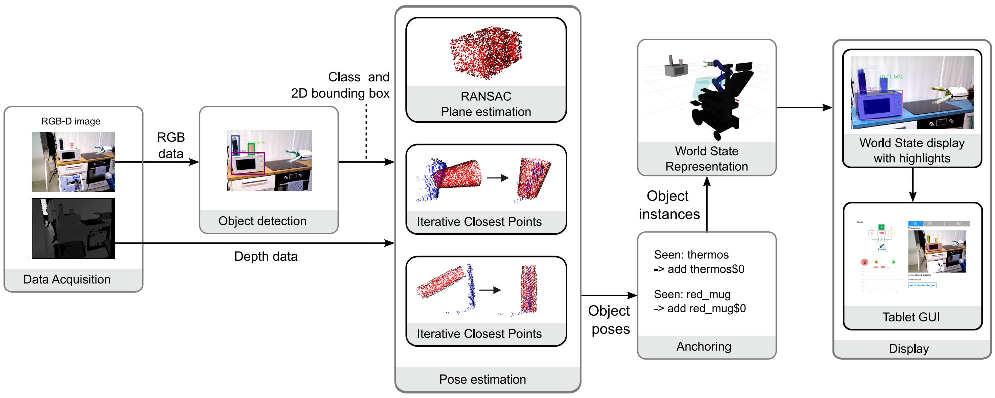

Fig. 8: **EDAN perception pipeline**. Example of the processing of an image with three object: a microwave, a red mug, and a bottle, with two different algorithms for pose estimation.

2.4.2 Object Detection

To be able to assist users in manipulating their environment, objects have to be identified and localized from the surroundings perceived by the robot, see also Figure 8. To do so, EDAN uses a RGB-D camera. Detection of the object is done from the ODB with an object detector, namely a fine-tuned RetinaNet with a ResNet 50 backbone and pretrained weights from the COCO dataset [12].

Let's denote $\mathcal{P}$ the inputs (RGB images from camera data) and $\mathcal{Y}$ the outputs (sets of object classes and their 2D bounding boxes), and define the object detector as a function $\mathcal{M}_\theta : \mathcal{P} \rightarrow \mathcal{Y}$ with parameters θ. For robustness, images of the objects of interest in a real scene were collected and annotated, denoted by $(\boldsymbol{p}_i, \boldsymbol{y}_i)_{i=1}^{Q}$. Q was around several hundreds (for a total of a dozen of objects) and can be tuned depending on the desired detection performance. With this annotated data set, the object detector was fine-tuned by optimizing on the parameters θ:

$$\theta^\star = \operatorname*{argmin}_\theta \frac{1}{Q} \sum_{i=1}^{Q} \mathcal{L}(\mathcal{M}_\theta(\boldsymbol{p}_i), \boldsymbol{y}_i)$$

where $\mathcal{L}(\cdot)$ is a loss function measuring the distance between predictions and ground truth outputs, which can be a cross entropy loss for classification or a mean square loss for regression.

As lightning conditions and object size can affect performance, we leveraged data augmentation and anchor-size optimization [13], which handle objects of different sizes better, to boost the performance. Furthermore, we attempted to replace real images of the training data – which require manual labeling – with synthetic counterparts to reduce the annotation efforts on preparing the training data set. The synthetic images were realized by recent advances of realistic image synthesizers [14] in combination with existing 3D object models. Having observed the sub-optimal performance due to the sim-to-real gap, we further took an initial step to devise a novel pipeline with Bayesian active learning to bridge the gap [15]. This saved annotation efforts for a subset of household objects used in the experiments such as the bottle, the red mug, the drawer handle and the door handle. Photo-realistic synthetic image generation worked due to these objects possessing 3D CAD models with faithful textures.

2.4.3 Object Localization

Once an object has been detected and its class identified, its 6D pose is estimated. First, the cropped depth data from the bounding box detection is converted into point cloud data. This point cloud data is then treated as the target of a registration method: the Iterative Closest Points (ICP) algorithm [16].

The source point cloud is created by loading the corresponding 3D object model from the ODB based on the predicted object class. The ICP algorithm then computes the rigid body transformation between the source and target point clouds, from which the pose of the object expressed in camera coordinate can be computed. To improve the matching precision, the ratio of outliers in the target point cloud is reduced by a series of operations such as plane segmentation and Euclidean clustering.

An alternative method dealt with tiny or thin objects like handles which rest on a vertical plane and yield undesirable performance with ICP. The vertical plane is estimated with the RANSAC algorithm [17] and the orientation of the object inferred based on the plane's

normal. The position of the object is then computed from the 3D coordinates of the point cloud.

2.4.4 Anchoring

Anchoring is defined by Coradeschi and Saffiotti as "*the problem of connecting, inside an artificial system, symbols and sensor data that refer to the same physical objects in the external world*" [18]. In the case of a mobile agent like EDAN, symbolic objects must retain consistence and persistence after robot actions: the anchoring process decides if after moving the wheelchair, an object seen from a different viewpoint is the same as before moving, or a different instance. Similarly, an object temporarily hidden by the manipulator keeps the same identifier.

To do so, an internal state tracks all localized objects, taking into consideration wheelchair odometry. A minimal distance is required between two anchored objects. In case of required arbitration (two different categories seen in the last few seconds with a similar pose for example), the quality of the pose as well as the number of detections in the last 5 seconds is taken into consideration. New detections of already anchored object update the object pose, while previously-known symbolic states, like a filled bottle status, can be maintained. If an object is new, an instance of its class is created in the WSR with a unique name. Finally, all existing world model objects not implicated in a task that are not perceived after 5 seconds are deleted from the WSR.

2.4.5 World State Representation

The WSR, also introduced by Leidner et. al. [11], contains the aggregated world model of the robot. As seen in Figure 7, it contains a list of all anchored objects and their tags, as well as their previously-known properties and the robot's belief in their current locations. It also keeps track of the symbolic properties of the objects. For example, after a grasping action, the *red_mug* would obtain a predicate *(grasped red_mug edan_arm)*.

The WSR serves as the information source for all other components in the shared control architecture. It is queried by other components like the Task Inference and the Shared Control Templates processes.

2.5 Task Inference

The task inference provides two functionalities: the first is identifying which shared control task can be executed based on the current world state; the second is automatically activating support once a task is likely enough. We make use of Planning Domain Definition Language (PDDL) to have skills with preconditions and effects, affecting the semantic state of the world. At 10 Hz, we evaluate which tasks are associated to the objects currently instantiated in the world, then select those with valid preconditions. The resulting list is displayed on the tablet GUI, from which the user can select at any time.

For each possible task, a distance from the EE to the target of the task, e.g. to the origin frame of the respective object is computed. Furthermore, the Active Constraints (ACs) of the first SCT-state are monitored by the task inference algorithm. As soon as the computed distance to the task is below a certain threshold as well as the ACs are fulfilled to a certain level, the task will start automatically.

2.6 Shared Control Templates

Assistance through shared control is implemented with SCTs [19]. SCTs help users move a robotic manipulator EE in task space to accomplish tasks. A template encodes how the user can move a target EE pose, which is tracked by the controller described in 2.3.5. The encoding constrains the user commanded velocity of the EE, as well as its pose.

A template is defined as a finite state machine. Therein, a state is a structure composed of robot parameters, PDDL effects, Input Mappings (IMs), and Active Constraints. Robot parameters can for example be the joint configuration of the tool, impedance controller parameters or thresholds for whole-body-control. We detail in the following subsections IMs, ACs and state transitions.

2.6.1 Input Mapping

An IM maps user velocity commands $\boldsymbol{u}$ to the EE velocity $\dot{\boldsymbol{x}}_{\text{sct}}$. Multiple IMs can be used consecutively, iteratively applying their displacements on $\boldsymbol{x}_{\text{sct}}$ during one time step. We denote $\boldsymbol{X}_{\text{sct}}$ the homogeneous transformation representation of $\boldsymbol{x}_{\text{sct}}$.

Formally, at each time step, an IM m first computes a displacement $\delta\boldsymbol{T}$:

$$\begin{aligned} \text{map}_m \colon \Re^n, SE(3) &\to SE(3) \\ \boldsymbol{u}(t) &\mapsto \delta\boldsymbol{T} \end{aligned}$$

then applies it on the desired pose of the EE:

$$\begin{aligned} \text{displace}_m \colon SE(3), SE(3) &\to SE(3) \\ \boldsymbol{X}_{\text{sct}}(t-1), \delta\boldsymbol{T} &\mapsto \boldsymbol{X}_{\text{im}}(t), \end{aligned}$$

with user command $\boldsymbol{u} \in \Re^n, n \in [\![1,6]\!]$ ($n = 3$ in this work). A displacement can be applied either in the local frame of reference, here the EE: $\text{displace}_m(\boldsymbol{X}_{\text{sct}}, \delta\boldsymbol{T}) = \boldsymbol{X}_{\text{sct}}\delta\boldsymbol{T}$ or the wheelchair frame of reference: $\text{displace}_m(\boldsymbol{X}_{\text{sct}}, \delta\boldsymbol{T}) = \delta\boldsymbol{T}\boldsymbol{X}_{\text{sct}}$.

To compute displacements, we make use of a Euler angle representation, adequate for the small rotations in a displacement. The most elementary IM is a 1-to-1 mapping, where each user input DoF controls one task space DoF. This can be computed with: $\text{map}_m = \text{euler\_to\_matrix}(\boldsymbol{A}\boldsymbol{u}), \boldsymbol{A} \in \Re^{6\times n}$, with euler_to_matrix a function to convert from a Euler representation to a homogeneous matrix representation and $\boldsymbol{A}$ a matrix with at most one element per line and per column.

An IM might not be directly computed on the EE, but can be applied on feature frames F_f, e. g. at the tip of a grasped bottle. Those feature frames can be static frames w. r. t. the EE, typically properties of a grasped object, saved in the Object Database. They can also be dynamically computed, e. g. at the tip of a bottle always pointing towards a target object. See Algorithm 1 for the implementation of an IM example using a 1-to-1 mapping.

A 1-to-1 mapping is not always the most intuitive manner to control a robot, in particular as soon as rotations are controlled by the user. For example, in the current implementation of *pour*, a scalar product is computed with 2 user input DoFs to control the tilting around the tip of a grasped bottle. Other features like dead-zones can also be used. This can provide a

Algorithm 1 Input Mapping

Input: User input $\boldsymbol{u}$, $\boldsymbol{X}_{\text{sct}}(t-1)$, Input Mapping IM
Output: Displaced target EE pose $\boldsymbol{X}_{\text{im}}(t)$
$\boldsymbol{F}_f \leftarrow$ IM.compute_feature_frame($\boldsymbol{X}_{\text{sct}}(t-1)$)
// Compute transform from $\boldsymbol{F}_f$ to $\boldsymbol{X}_{\text{sct}}(t-1)$
${}_{F_f}\boldsymbol{T}^{X_{\text{sct}}} \leftarrow \boldsymbol{F}_f{}^{-1}\boldsymbol{X}_{\text{sct}}(t-1)$
// Compute displacement
$\delta\boldsymbol{T} \leftarrow$ euler_to_matrix($\boldsymbol{A}\boldsymbol{u}$)
// If Local: Apply local displacement to the feature frame
$\boldsymbol{F}^{\text{displaced}} \leftarrow \boldsymbol{F}_f \delta\boldsymbol{T}$
// Update target EE pose from the new feature frame
$\boldsymbol{X}_{\text{im}}(t) \leftarrow \boldsymbol{F}^{\text{displaced}}{}_{F_f}\boldsymbol{T}^{X_{\text{sct}}}$
return $\boldsymbol{X}_{\text{im}}(t)$

reduction of the number of controlled DoF w. r. t. the dimension of the user input, make the IM more intuitive for the user, and adapt to user preferences.

2.6.2 Active constraints

An AC restricts the space of permissible poses in task space. Complementary to an IM which applies on velocities, an AC acts on positions.

An AC m is implicitly defined by its projection function:

$$\begin{aligned} \text{project}_m \colon SE(3) &\rightarrow SE(3) \\ \boldsymbol{X}_{\text{im}}(t) &\mapsto \boldsymbol{X}_{\text{ac}}(t), \end{aligned}$$

Multiple ACs can be active within the same state, and are applied iteratively. As with IM models, an AC can be applied on feature frames F_f. Changes on F_f are then reflected back on a new $\boldsymbol{X}_{\text{ac}}$ pose, cf Algorithm 2.

Algorithm 2 Active Constraint

Input: $\boldsymbol{X}_{\text{im}}(t)$, Active Constraint AC
Output: Constrained target EE pose $\boldsymbol{X}_{\text{ac}}(t)$
$\boldsymbol{X}_{\text{ac}} \leftarrow \boldsymbol{X}_{\text{im}}(t)$
$\boldsymbol{F}_f \leftarrow$ AC.compute_feature_frame($\boldsymbol{X}_{\text{ac}}$)
${}_{F_f}\boldsymbol{T}^{X_{\text{ac}}} \leftarrow \boldsymbol{F}_f^{-1}\boldsymbol{X}_{\text{ac}}$
// Apply constraints on the feature frame
$\boldsymbol{F}^{\text{projected}} \leftarrow$ AC.project$_m(\boldsymbol{F}_f)$
// Update target EE pose from feature frame
$\boldsymbol{X}_{\text{ac}} \leftarrow \boldsymbol{F}^{\text{projected}}{}_{F_f}\boldsymbol{T}^{X_{\text{ac}}}$
return $\boldsymbol{X}_{\text{ac}}$

An AC can for example constrain the EE position within a plane, or within a cone to guide the EE towards a grasp frame, or set the EE orientation based on the EE position when grasping a cylindrical object.

Some constraints can be represented by either an IM or an AC: for example, moving along a circle. Assuming that at t_0 the EE is already located on the circle, an IM can be defined as the tangent direction along this circle. With an ideal discretization, this will restrict the user to only move along this circle. However, one could also allow free motion with the IM, then use an AC to project $\boldsymbol{X}_{\mathrm{ac}}$ onto the circle. The resulting set of poses the EE can get to is the same in both cases: there is no unique template definition. In practice, the choice of constraining via IMs or ACs depends on the resulting intuitiveness of use and the complexity required to define the template.

2.6.3 Finite State Machine

An SCT consists of multiple states, described above. Together with transitions, they form a finite state machine. There is a defined start state q_0 for when the task is started and there can only be one active state at a time, described in [20]. The state machine additionally defines a distinct end state q_{end}. If this state is reached, the execution of a task finishes.

Transitions cause a state change within an SCT, if a corresponding event is triggered. Events can be spatial conditions, for example the crossing of a certain threshold of some measure, like the Euclidean distance from EE to an object. It can also be crossing a threshold of force interaction, e. g. creating contact with a drawer, or sensor values such as a time-of-flight sensor in the EE detecting the presence of an object, before closing the fingers. Temporal conditions as events are seldom used, to allow the user to execute a task at their own rhythm.

2.6.4 Trajectories of tasks execution

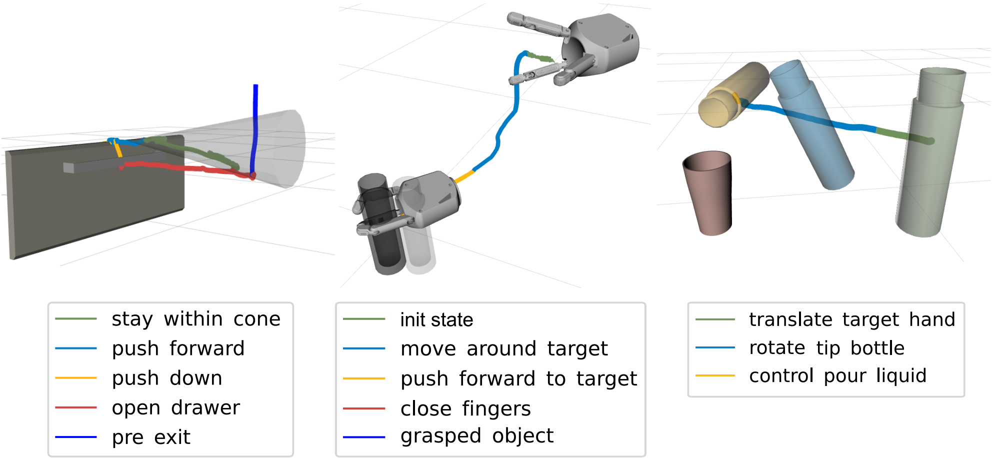

Fig. 9: **End effector measured trajectories for the tasks *open drawer*, *pick* and *pour* executed by the participants.** The different states of the finite state machine of the used shared control template are shown.

An illustration of the result of an SCT execution is shown in Figure 9. The participants could for example select the approach angle to pick objects or pour, as well as the tilt angle when pouring; see also the attached videos.

References

[1] Fitts, P. M. The information capacity of the human motor system in controlling the amplitude of movement. *Journal of experimental psychology* **47**, 381 (1954).

[2] Hudgins, B., Parker, P. & Scott, R. N. A new strategy for multifunction myoelectric control. *IEEE Transactions on Biomedical Engineering (TBME)* **40**, 82–94 (1993).

[3] Vogel, J. & Hagengruber, A. An sEMG-based interface to give people with severe muscular atrophy control over assistive devices. *2018 40th Annual International Conference of the IEEE Engineering in Medicine and Biology Society (EMBC)* 2136–2141 (2018).

[4] Neumann, M., Huang, S., Marthaler, D. E. & Kersting, K. pygps: a python library for gaussian process regression and classification. *The Journal of Machine Learning Research (JMLR)* **16**, 2611–2616 (2015).

[5] Rasmussen, C. E. & Nickisch, H. Gaussian processes for machine learning (gpml) toolbox. *The Journal of Machine Learning Research (JMLR)* **11**, 3011–3015 (2010).

[6] Albu-Schäffer, A., Ott, C. & Hirzinger, G. A unified passivity-based control framework for position, torque and impedance control of flexible joint robots. *International Journal of Robotics Research (IJRR)* **26**, 23–39 (2007).

[7] Iskandar, M. *et al.* Joint-level control of the DLR lightweight robot SARA. *2020 IEEE/RSJ International Conference on Intelligent Robots and Systems (IROS)* 8903–8910 (2020).

[8] Iskandar, M., Eiberger, O., Albu-Schäffer, A., De Luca, A. & Dietrich, A. Collision detection, identification, and localization on the DLR SARA robot with sensing redundancy. *2021 IEEE International Conference on Robotics and Automation (ICRA)* 3111–3117 (2021).

[9] Shoemake, K. Animating rotation with quaternion curves. *Proceedings of the 12th annual conference on Computer graphics and interactive techniques (SIGGRAPH)* 245–254 (1985).

[10] Weitschat, R., Dietrich, A. & Vogel, J. Online motion generation for mirroring human arm motion. *2016 IEEE International Conference on Robotics and Automation (ICRA)* 4245–4250 (2016).

[11] Leidner, D., Borst, C. & Hirzinger, G. Things are made for what they are: Solving manipulation tasks by using functional object classes. *2012 IEEE International Conference on Humanoid Robots (HUMANOIDS)* 429–435 (2012).

[12] Lin, T.-Y. *et al.* Microsoft coco: Common objects in context. *European conference on computer vision (ECCV)* 740–755 (2014).

[13] Zlocha, M., Dou, Q. & Glocker, B. Improving retinanet for ct lesion detection with dense masks from weak recist labels. *International conference on medical image computing and computer-assisted intervention (MICCAI)* 402–410 (2019).

[14] Denninger, M. *et al.* Blenderproc. *arXiv preprint, arXiv:1911.01911* (2019).

[15] Feng, J., Lee, J., Durner, M. & Triebel, R. Bayesian active learning for sim-to-real robotic perception. *2022 IEEE/RSJ International Conference on Intelligent Robots and Systems (IROS)* (2022).

[16] Besl, P. J. & McKay, N. D. *Method for registration of 3-d shapes*, Vol. 1611, 586–606 (Spie, 1992).

[17] Fischler, M. A. & Bolles, R. C. Random sample consensus: a paradigm for model fitting with applications to image analysis and automated cartography. *Communications of the ACM* **24**, 381–395 (1981).

[18] Coradeschi, S. & Saffiotti, A. An introduction to the anchoring problem. *Robotics and Autonomous Systems* **43**, 85–96 (2003).

[19] Quere, G. *et al.* Shared control templates for assistive robotics. *2020 IEEE International Conference on Robotics and Automation (ICRA)* 1956–1962 (2020).

[20] Hopcroft, J. E., Motwani, R. & Ullman, J. D. Introduction to automata theory, languages, and computation. *Acm Sigact News* **32**, 60–65 (2001).
